# Author Correction: Myotonic dystrophy RNA toxicity alters morphology, adhesion and migration of mouse and human astrocytes

**DOI:** 10.1038/s41467-022-31774-7

**Published:** 2022-07-14

**Authors:** Diana M. Dincã, Louison Lallemant, Anchel González-Barriga, Noémie Cresto, Sandra O. Braz, Géraldine Sicot, Laure-Elise Pillet, Hélène Polvèche, Paul Magneron, Aline Huguet-Lachon, Hélène Benyamine, Cuauhtli N. Azotla-Vilchis, Luis E. Agonizantes-Juárez, Julie Tahraoui-Bories, Cécile Martinat, Oscar Hernández-Hernández, Didier Auboeuf, Nathalie Rouach, Cyril F. Bourgeois, Geneviève Gourdon, Mário Gomes-Pereira

**Affiliations:** 1 Sorbonne Université, Inserm, Centre de Recherche en Myologie, 75013 Paris, France; 2 Neuroglial Interactions in Cerebral Physiology and Pathologies, Center for Interdisciplinary Research in Biology, Collège de France, CNRS, Inserm, Labex Memolife, 75005 Paris, France; 3 Inserm UMR1163, Institut Imagine, Université Paris Cite, 75015 Paris, France; 4 Doctoral School N°562, Paris Descartes University, Paris, 75006 France; 5 Inserm/UEVE UMR861, Université Paris Saclay I-STEM, 91110 Corbeil-Essonnes, France; 6 Laboratory of Genomic Medicine, Department of Genetics, National Rehabilitation Institute (INR-LGII), Mexico City, Mexico; 7 Laboratoire de Biologie et Modelisation de la Cellule, Ecole Normale Superieure de Lyon, CNRS, UMR 5239, Inserm, U1293, Universite Claude Bernard Lyon 1, 46 allée d’Italie, 69364 Lyon, France

**Keywords:** Diseases of the nervous system, Focal adhesion, Astrocyte

Correction to: *Nature Communications* 10.1038/s41467-022-31594-9, published online 04 July 2022.

In this article the author name Julie Tahraoui-Bories was incorrectly written as Julie Tahraoui-Boris. The original article has been corrected.

