# Supplementary material for: Myotonic dystrophy RNA toxicity alters morphology, adhesion and migration of mouse and human astrocytes
Source: Nat Commun. 2022 Jul 4;13:3841. doi: 10.1038/s41467-022-31594-9 (PMC9253038; doi:10.1038/s41467-022-31594-9)
Supplement: Supplementary file 1 — Supplementary Information [file 41467_2022_31594_MOESM1_ESM.pdf]

Supplementary Information

Myotonic dystrophy RNA toxicity alters astrocyte morphology, adhesion and migration

Diana M. Dincã, Louison Lallemant, Anchel Gonzalez-Barriga, Noémie Cresto, Sandra O. Braz, Géraldine Sicot, Laure-Elise Pillet, Hélène Polveche, Paul Magneron, Aline Huguet-Lachon, Hélène Benyamine, Cuauhtli N. Azotla-Vilchis, Luis E. Agonizantes-Juárez, Julie Tahraoui, Cécile Martinat, Oscar Hernández-Hernández, Didier Auboeuf, Nathalie Rouach, Cyril F. Bourgeois, Geneviève Gourdon, Mário Gomes-Pereira

- Supplementary Fig. 1
- Supplementary Fig. 2
- Supplementary Fig. 3
- Supplementary Fig. 4
- Supplementary Fig. 5
- Supplementary Fig. 6
- Supplementary Fig. 7
- Supplementary Fig. 8
- Supplementary Fig. 9
- Supplementary Fig. 10
- Supplementary Fig. 11
- Supplementary Table 1
- Supplementary Table 2
- Supplementary Table 3
- Supplementary Table 4
- Supplementary Table 5
- Supplementary Table 6
- Supplementary Table 7
- Supplementary References
- Original uncropped scans of blots and gels

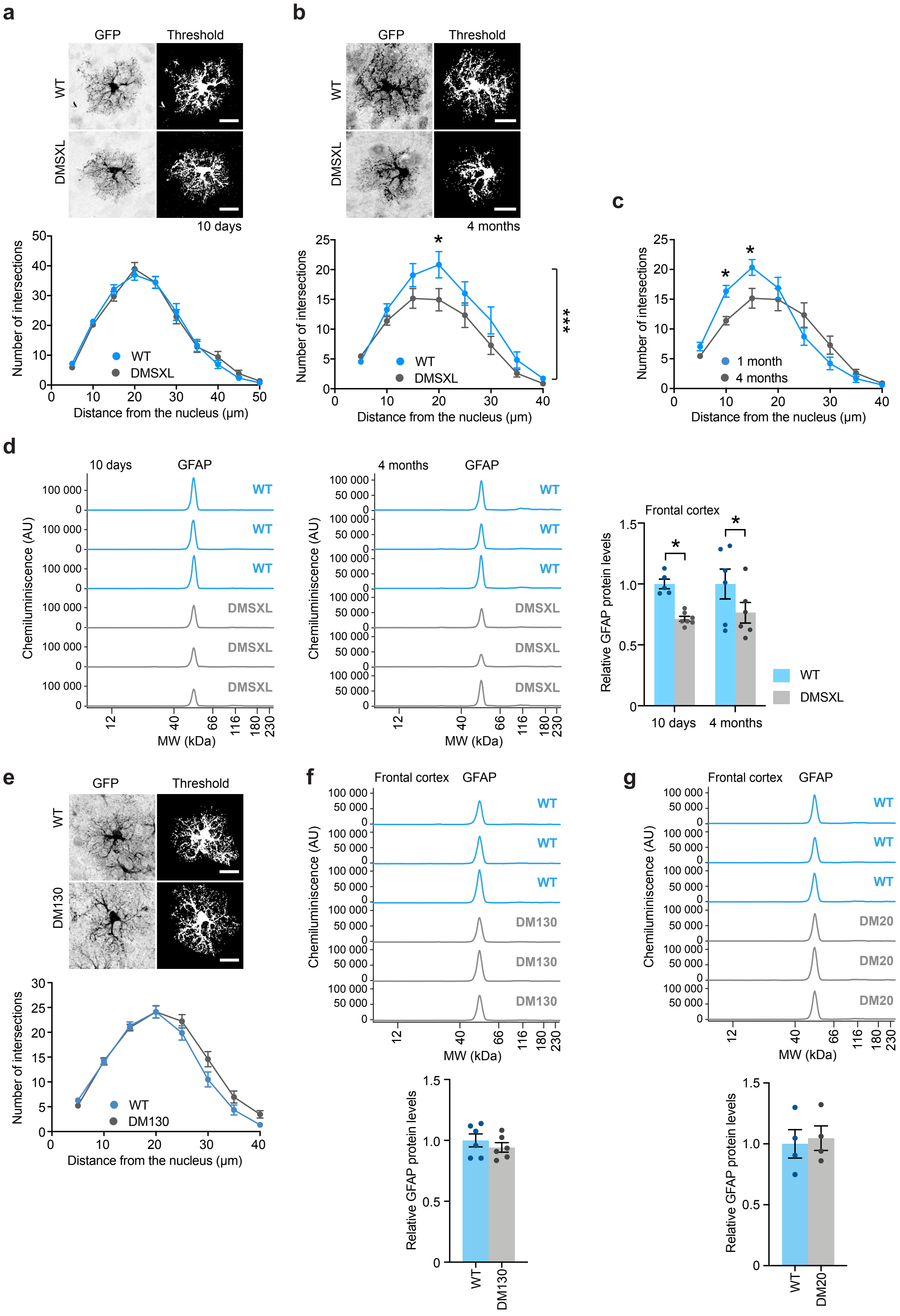
a
GFP
Threshold
WT
DMSXL
10 days
Number of intersections
Distance from the nucleus (µm)
WT
DMSXL
b
GFP
Threshold
WT
DMSXL
4 months
*
***
c
*
*
1 month
4 months
d
10 days
GFAP
Chemiluminiscence (AU)
MW (kDa)
4 months
Frontal cortex
Relative GFAP protein levels
e
DM130
f
g
DM20

Supplementary Fig. 1. Astrocyte morphology and GFAP expression. (a) Sholl analysis of the branching of DMSXL cortical astrocytes at postnatal day 10. Scale bar, 20 µm. Data are means ± SEM. N = 4 mice, n = 20 cells, WT; N = 4 mice, n = 20 cells, DMSXL (p = 0.9890, Two-way ANOVA). **(b)** Sholl analysis of the branching of DMSXL cortical astrocytes at 4 months. Scale bar, 20 µm. Data are means ± SEM. N = 4 mice, n = 17 cells, WT; N = 4 mice, n = 18 cells, DMSXL (p = 0.0002, Two-way ANOVA; p = 0.0195, 20 µm; Sidak post-hoc test for multiple comparisons). **(c)** Comparison of cortical DMSXL astrocyte branching at 1 and 4 months. Data are means ± SEM. N = 4 mice, n = 20 cells, 1 month; N = 4 mice, n = 18 cells, 4 months (p = 0.0390, 10 µm; p = 0.0264, 15 µm; Two-way ANOVA, Sidak post-hoc test for multiple comparisons). **(d)** Quantification of GFAP expression in whole frontal cortex tissue lysates of control and DMSXL mice at 10 days and 4 months of age. Representative electrophoretic profiles are shown on the left. Data are means ± SEM. 10 days: n = 4, WT; n = 4, DMSXL. 4 months: n = 6, WT; n = 6, DMSXL (p = 0.0177, 10 days; p = 0.0426, 4 months; Two-way ANOVA, Sidak post-hoc test for multiple comparisons). **(e)** Sholl analysis of the branching of DM130 cortical astrocytes at 1 month. GFP signal was analyzed by applying the same threshold to all images. Scale bar, 20 µm. Data are means ± SEM. N = 4 mice, n = 26 cells, WT; N = 4 mice, n = 27 cells, DMSXL (p = 0.1141, Two-way ANOVA). **(f)** Quantification of GFAP expression in whole frontal cortex tissue lysates of control DM130 mice at 1 month, by capillary basis electrophoresis. Representative electrophoretic profiles are shown. Data are means ± SEM. n = 6, WT; n = 6, DM130 (p=0.3972, Two-tailed Student's *t* test). **(g)** Quantification of GFAP expression in whole frontal cortex tissue lysates of control DM20 mice, at 1 month. Representative electrophoretic profiles are shown. Data are means ± SEM. n = 4, WT; n = 4, DM20 (p=0.7729, Two-tailed Student's *t* test). Source data are provided as a Source Data file. * p<0.05; *** p<0.001.

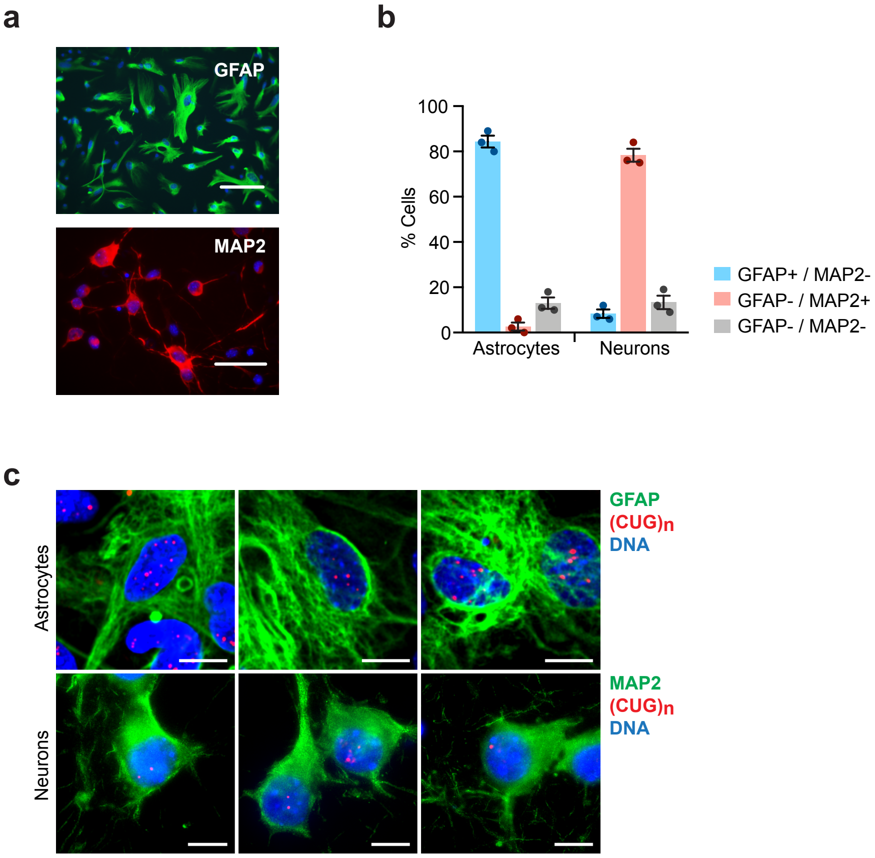

Supplementary Fig. 2. Expression of astrocytic and neuronal markers in primary cell cultures derived from mouse brain cortex. (a) We confirmed astrocyte and neuron enrichment in primary cultures by the immunofluorescence of astrocyte- and neuron-specific proteins, GFAP and MAP2, respectively. Representative immunofluorescent detection of primary mouse astrocytes expressing GFAP (green), and primary mouse neurons expressing MAP2 (red). Scale bar, 100 µm. The experiment was performed on 3 independent cultures of each cell type. **(b)** Percentage of primary cells expressing GFAP or MAP2. Data are means ± SEM; n = 3 independent WT cultures. The expression analysis of GFAP and MAP2 revealed that 84.3 ± 4.5% of primary cells growing under conditions that favor astrocyte proliferation expressed GFAP. Conditions that favor neuronal survival yielded MAP2 staining in 78.3 ± 4.9 % of cells. **(c)** FISH detection of RNA foci in primary DMSXL mouse brain cells, illustrating a higher number of nuclear aggregates in GFAP-expressing astrocytes than in MAP2-expressing neurons. Scale bar, 10 µm. The experiment was performed on 3 independent cultures of each cell type. Source data are provided as a Source Data file.

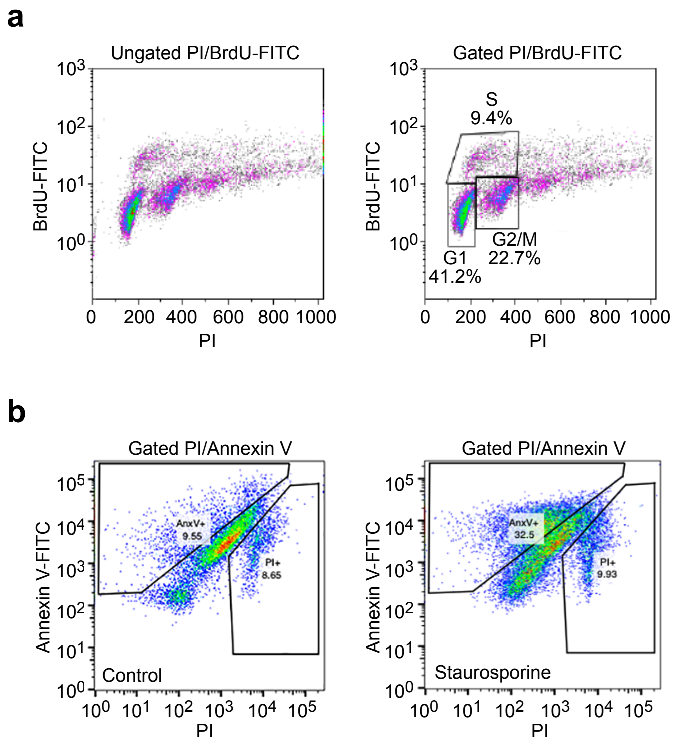

Supplementary Fig. 3. FACS gating strategies. (a) Example of flow cytometric gating for the quantification of distribution of cultured astrocytes between different phases of cell cycle. Cells were gated for PI and BrdU-FITC. **(b)** Example of flow cytometric gating for the quantification of apoptosis, under control conditions and staurosporine-induced stress. Cells were gated for PI and Annexin V-FITC.

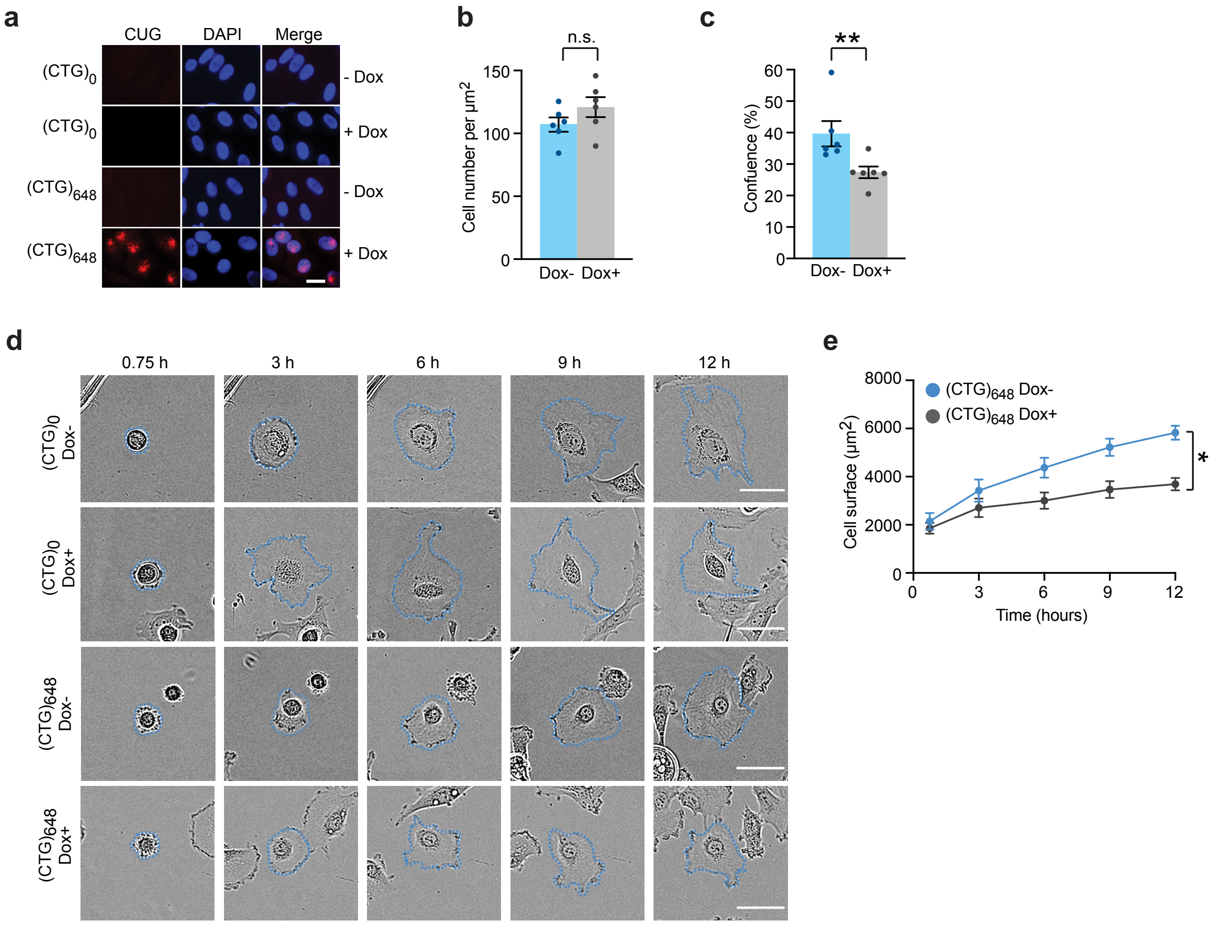

Supplementary Fig. 4. Human glial cells transfected with expanded CTG repeats exhibit abnormal adhesion and reduced cell size. (a) FISH detection of CUG RNA foci in human glia MIO-M1 cells expressing an inducible $(CTG)_{648}$ *DMPK* RNA, or a no-expansion $(CTG)_0$ control construct. Transgene induction by doxycycline was performed over 3 days and is indicated (Dox+, Dox-). Scale bar, 10 µm. The experiment was performed on 5 independent cultures of each group. **(b)** Quantification of the number of MIO-M1 cells attached 45 minutes after plating. Data are means ± SEM, n = 5 independent cultures per treatment (p = 0.1811, Two-tailed Student's *t* test). **(c)** Semi-automated quantification of cell culture confluence 45 minutes after plating. Data are means ± SEM, n = 5 independent cultures per treatment (p = 0.0087, Two-tailed Mann-Whitney *U* test). **(d)** Representative time-lapse bright field images of glial MIO-M1 cells expressing normal or expanded *DMPK* transcripts, over 12 hours after plating. Scale bar, 50 µm. The experiment was performed on 5 independent cultures of each group. **(e)** Size of individual MIO-M1 $(CTG)_{648}$ glial cells, following transgene induction (Dox+), versus non-induced cells (Dox-). Data are means ± SEM, n = 31 cells, Dox+; n = 30 cells, Dox- (p = 0.0246, Two-way repeated measures ANOVA). Source data are provided as a Source Data file. n.s., not significant; * p<0.05; ** p<0.01.

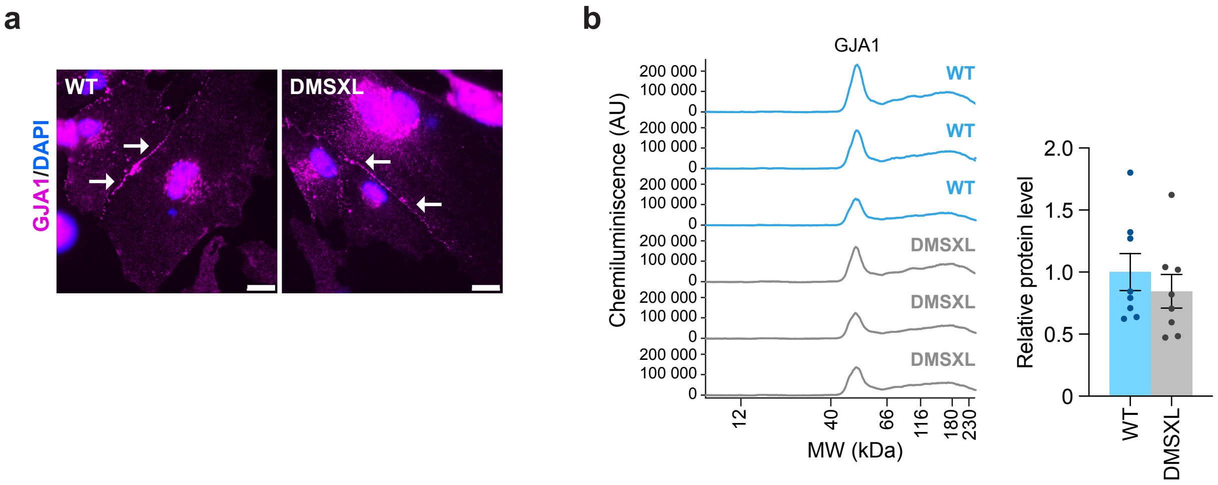

Supplementary Fig. 5. Primary DMSXL astrocytes do not show abnormal assembly of gap junctions or altered expression GJA1 proteins. (a) Immunofluorescence of GJA1 (magenta) showing punctuated staining of gap junctions (white arrows) along areas of cell-to-cell contact in cultured primary astrocytes. Scale bar, 20 µm. The experiment was performed on 3 independent cultures of each genotype. **(b)** Quantification of GJA1 protein levels in primary astrocytes by capillary basis electrophoresis. Representative electrophoretic profiles are shown on the left. Data are means ± SEM. n = 7 independent cultures per genotype ($p = 0.2680$, Two-tailed Student's *t* test). Source data are provided as a Source Data file.

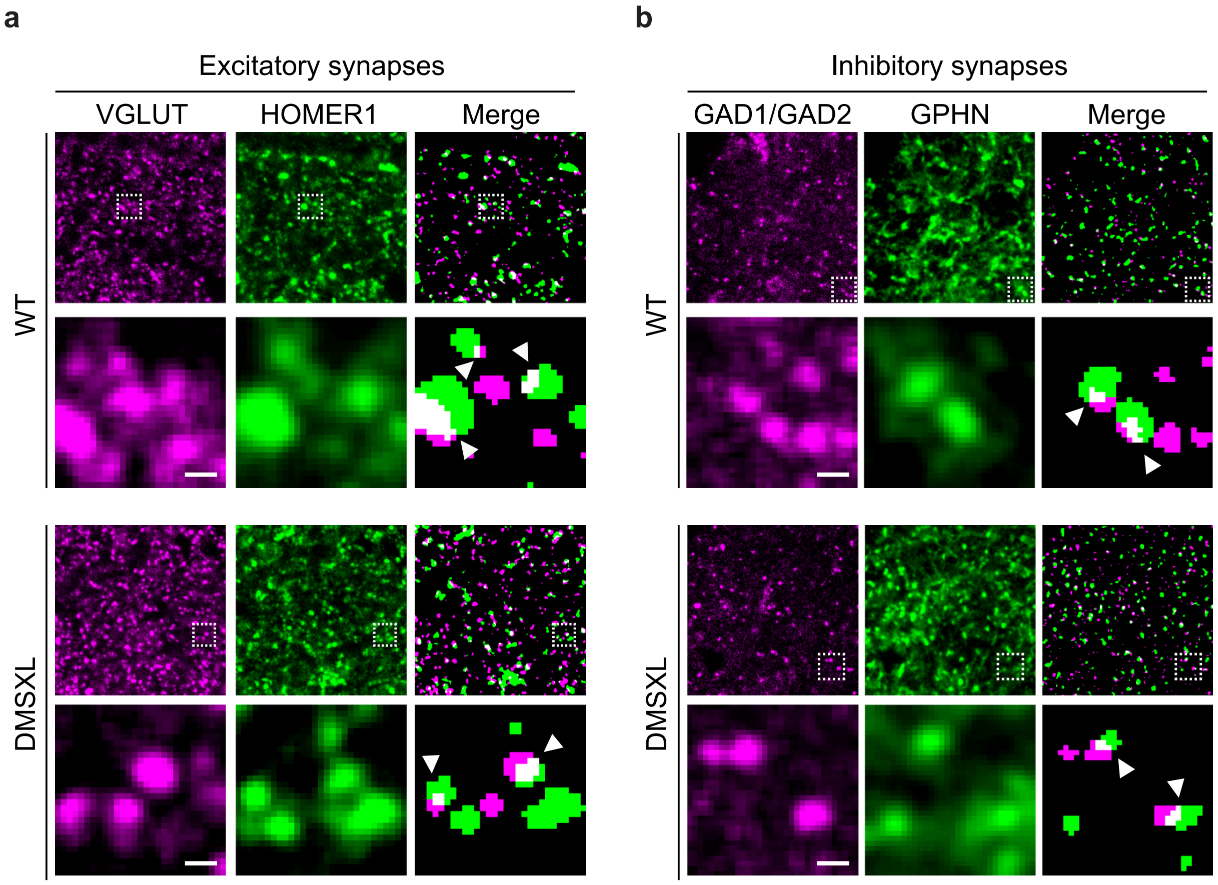

Supplementary Fig. 6. Visualization of excitatory and inhibitory synapses in mouse frontal cortex. (a) Excitatory synapses were identified by the co-localized clusters (merge, white) of VGLUT1 (magenta) and HOMER1 (green). **(b)** Inhibitory synapses were identified by the co-localized clusters (merge, white) of GAD1/GAD2 (magenta) and gephyrin (GPHN, green). High magnification insets show co-localization analysis (white arrowheads) performed on binary images (masks). Scale bar, 5 µm. The experiments were performed on 5 independent animals of each cell type.

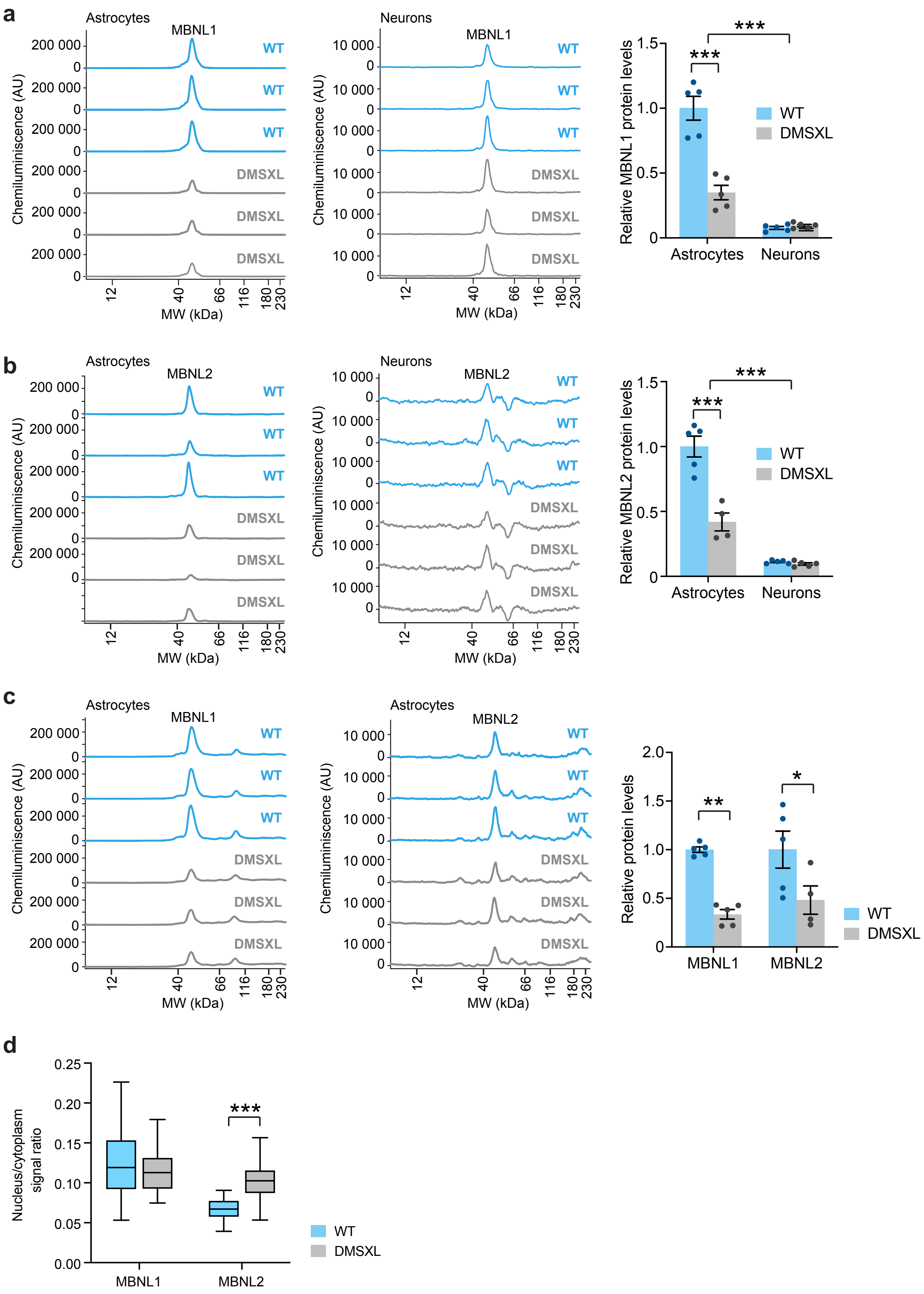
a
Astrocytes
MBNL1
Neurons
MBNL1
WT
DMSXL
Chemiluminiscence (AU)
MW (kDa)
Relative MBNL1 protein levels
Astrocytes
Neurons
b
Astrocytes
MBNL2
Neurons
MBNL2
Relative MBNL2 protein levels
c
Astrocytes
MBNL1
Astrocytes
MBNL2
Relative protein levels
MBNL1
MBNL2
d
Nucleus/cytoplasm signal ratio
MBNL1
MBNL2
WT
DMSXL

Supplementary Fig. 7. Primary DMSXL astrocytes show MBNL1 and MBNL2 protein downregulation. (a) Quantification of MBNL1 protein levels in primary astrocytes and neurons by capillary basis electrophoresis, using an antibody against recombinant full length MBNL1 protein. Representative electrophoretic profiles are shown on the left. Data are means ± SEM, n = 5 independent cell cultures per genotype ($p<0.0001$, Two-way ANOVA; $p<0.0001$, astrocytes, Sidak post-hoc pair-wise comparisons). **(b)** Quantification of MBNL2 protein levels in primary astrocytes and neurons by capillary basis electrophoresis, using an antibody raised against recombinant full length MBNL2 protein. Representative electrophoretic profiles are shown on the left. Data are means ± SEM, n = 5 independent cell cultures per genotype ($p<0.0001$, Two-way ANOVA; $p<0.0001$, astrocytes, Sidak post-hoc pair-wise comparisons). **(c)** Quantification of MBNL1 and MBNL2 protein levels in primary astrocytes and neurons by capillary basis electrophoresis, using independent antibodies raised against synthetic peptides corresponding to amino acid sequences 250-350 and 46-95 of human proteins, respectively. Representative electrophoretic profiles are shown on the left. Data are means ± SEM. MBNL1: n = 5 independent astrocyte cultures per genotype. MBNL2: n = 5 independent WT astrocyte cultures, n = 4 independent DMSXL astrocyte cultures ($p = 0.0024$, MBNL1; $p = 0.0209$, MBNL2, Two-way ANOVA, Sidak post-hoc pair-wise comparisons). **(d)** Nucleo-cytoplasmic localization of MBNL1 and MBNL2 in primary astrocytes. Tukey whisker plots represent the ratio between the nuclear and cytoplasmic immunofluorescent signal. The plots display the median and extend from the 25th percentile up to the 75th percentiles. The whiskers are drawn down to the 10th percentile, and up to the 90th percentile. N = 5 independent cultures per genotype. MBNL1: n = 38 cells, WT; n = 36 cells, DMSXL. MBNL2: n = 26 cells, WT; n = 44 cells, DMSXL ($p<0.0001$, Two-way ANOVA, Sidak post-hoc pair-wise comparisons). Source data are provided as a Source Data file. * $p<0.05$; ** $p<0.01$; *** $p<0.001$.

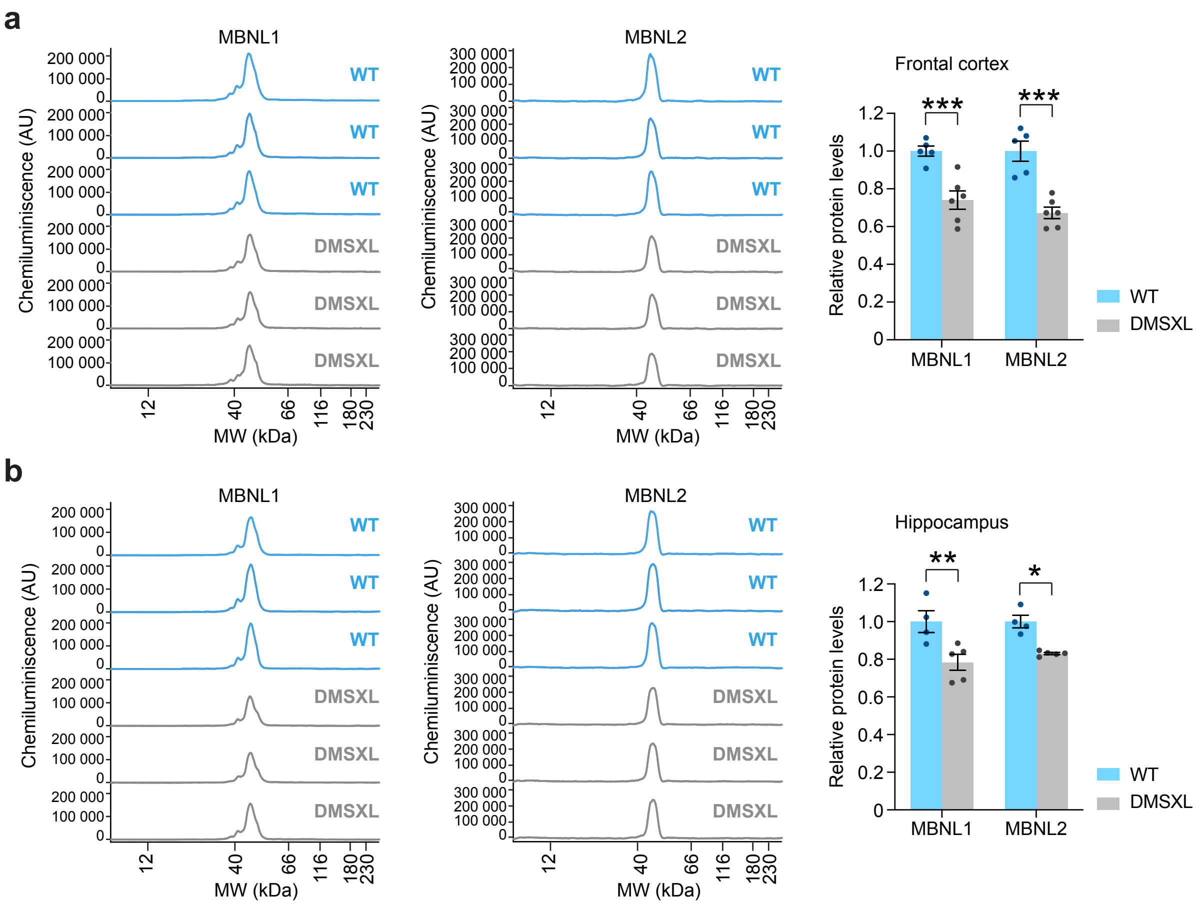

Supplementary Fig. 8. DMSXL frontal cortex and hippocampus show MBNL1 and MBNL2 protein downregulation. (a) Quantification of MBNL1 and MBNL2 protein levels in whole mouse frontal cortex tissue lysates at 1 month capillary basis electrophoresis, using antibodies against recombinant full length human proteins. Representative electrophoretic profiles are shown on the left. Data are means ± SEM; n = 5, WT; n = 6, DMSXL ($p = 0.0007$, MBNL1, $p<0.0001$, MBNL2; Two-way ANOVA, Sidak post-hoc pair-wise comparisons). **(b)** Quantification of MBNL1 and MBNL2 protein levels in whole mouse hippocampus tissue lysates at 1 month by capillary basis electrophoresis, using antibodies raised against recombinant full length human proteins. Representative electrophoretic profiles are shown on the left. Data are means ± SEM, n = 4, WT; n = 5, DMSXL ($p = 0.0026$, MBNL1; $p = 0.0143$, MBNL2; Two-way ANOVA, Sidak post-hoc pair-wise comparisons). Source data are provided as a Source Data file. * $p<0.05$; ** $p<0.01$; *** $p<0.001$.

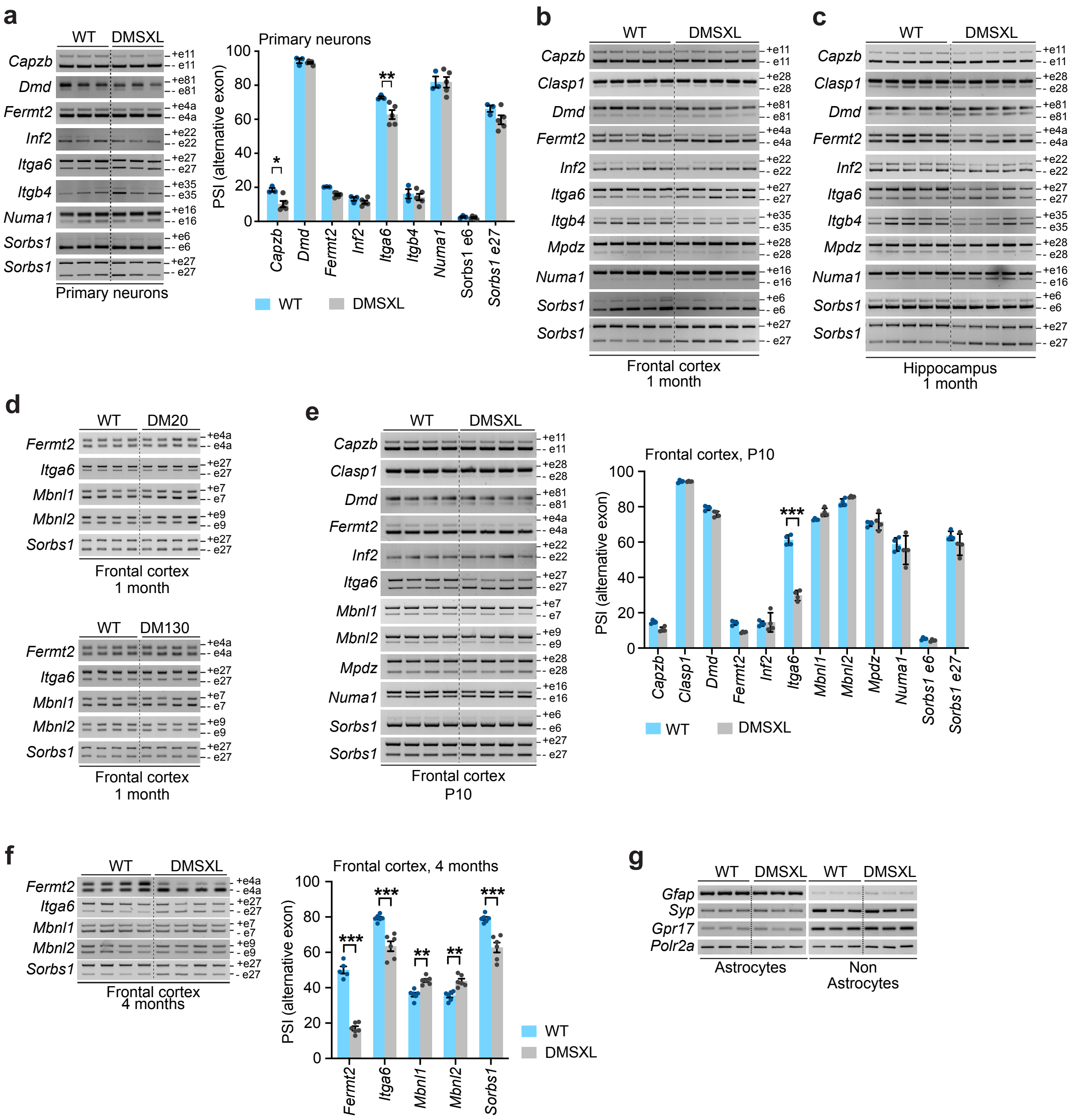

Supplementary Fig. 9. DM1 RNA toxicity is more pronounced in mouse astrocytes than in neurons. (a) Representative RT-PCR splicing analysis of transcripts associated with cell adhesion and cytoskeleton in primary DMSXL and WT neurons. Alternative exons are indicated on the right. The graph shows the PSI quantification of alternative exons. Data are means ± SEM, n = 3 independent WT cultures, n = 5 independent DMSXL cultures ($p = 0.0394$, *Capzb*; $p > 0.9999$, *Dmd*, *Numa1*, *Sorbs1* e6; $p = 0.6190$, *Fermt2*; $p = 0.9984$, *Inf2*; $p = 0.0066$, *Itga6*; $p = 0.9970$, *Itgb4*; $p = 0.1919$, *Sorbs1* e27; Two-way ANOVA, Sidak post-hoc test for multiple comparisons). **(b)** Representative RT-PCR analysis of *Capzb, Clasp1*, *Dmd, Fermt2, Inf2, Itga6, Itgb4, Mpdz, Numa1, Sorbs1* in DMSXL frontal cortex, relative to WT control mice at 1 month of age. Alternative exons are indicated on the right. The experiment was performed on 4-5 animals of each genotype. **(c)** Representative RT-PCR splicing analysis of *Capzb, Clasp1*, *Dmd, Fermt2, Inf2, Itga6, Itgb4, Mpdz, Numa1, Sorbs1* in DMSXL hippocampus, relative to WT control mice at 1 month of age. Alternative exons are indicated on the right. The experiment was performed on 6 animals of each genotype.

(d) Representative RT-PCR splicing analysis of *Fermt2, Itga6, Mbnl1, Mbnl2* and *Sorbs1* in the frontal cortex of 1-month-old DM20 and DM130 mice, expressing shorter CUG RNA transcripts. Alternative exons are indicated on the right. The experiment was performed on 4 animals of each genotype. **(e)** Representative RT-PCR splicing analysis of selected transcripts in the frontal cortex of DMSXL and WT mice at postnatal 10. Alternative exons are indicated on the right. PSI quantification of alternative exons. Data are means ± SEM, n = 4 mice per genotype (p = 0.4781, *Capzb*; p > 0.9999, *Clasp1*, *Inf2*, *Mpdz*, *Sorbs1* e6; p = 0.7121, *Dmd*; p = 0.1563, *Fermt2*; p < 0.0001, *Itga6*; p = 0.5083, *Mbnl1*; p = 0.8247, *Mbnl2*; p = 8181, *Numa1*; p = 0.2244, *Sorbs1* e27; Two-way ANOVA, Sidak post-hoc test for multiple comparisons). **(f)** Representative RT-PCR splicing analysis in the frontal cortex of DMSXL mice at 4 months of age. Alternative exons are indicated on the right. The graph shows the PSI quantification of alternative exons. Data are means ± SEM, n = 5-6 mice per genotype (p < 0.0001, *Fermt2*, *Itga6*, *Sorbs1*; p = 0.0058, *Mbnl1*; p = 0.0036, *Mbnl2*; Two-way ANOVA, Sidak post-hoc test for multiple comparisons). **(g)** Semi-quantitative expression analysis of *Gfap*, *Syp* and *Gpr17* in astrocyte and non-astrocyte cell fractions, purified from the forebrain of 1-month-old mice. n = 3 independent cell fractions, collected from 3 animals per genotype. Source data are provided as a Source Data file. * p<0.05; ** p<0.01; *** p<0.001.

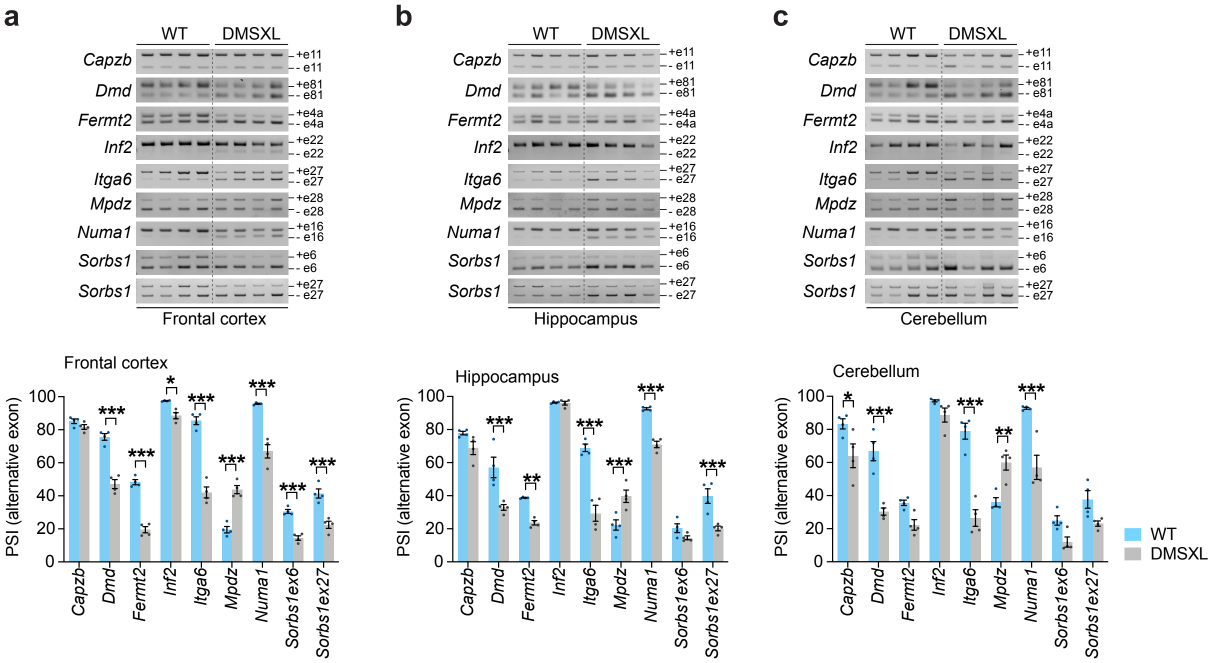

Supplementary Fig. 10. Regional distribution of RNA missplicing in astrocytes isolated from different brain areas (a) Representative RT-PCR splicing analysis in astrocytes acutely isolated from the frontal cortex of 1-month-old mice (p = 0.9363, *Capzb*; p < 0.0001, *Dmd*, *Fermt2*, *Itga6*, *Mpdz*, *Numa1*, *Sorbs1* e6, *Sorbs1* e27, p = 0.0385, *Inf2*; Two-way ANOVA, Sidak post-hoc test for multiple comparisons). **(b)** Representative RT-PCR splicing analysis in astrocytes acutely isolated from the hippocampus of 1-month-old mice (p = 0.2313, *Capzb*; p < 0.0001, *Dmd*, *Itga6*, *Numa1*, *Sorbs1* e27, p = 0.0040, *Fermt2*; p > 0.9999, *Inf2*; p = 0.0006, *Mpdz*; p = 0.7837, *Sorbs1* e6; Two-way ANOVA, Sidak post-hoc test for multiple comparisons). **(c)** Representative RT-PCR splicing analysis in astrocytes acutely isolated from the cerebellum of 1-month-old mice (p = 0.0159, *Capzb*; p < 0.0001, *Dmd*, *Itga6*, *Numa1*; p = 0.2146, *Fermt2*; p = 0.7672, *Inf2*; p = 0.0013, *Mpdz*; p = 0.2406, *Sorbs1* e6; p = 0.1338, *Sorbs1* e27; Two-way ANOVA, Sidak post-hoc test for multiple comparisons). Alternative exons are indicated on the right. The graphs represent the PSI quantification of alternative exons in astrocytes acutely isolated from individual brain areas. Data are means ± SEM, n = 4 independent cell fractions per genotype, isolated from 4 different animals. Source data are provided as a Source Data file. * p<0.05; ** p<0.01; *** p<0.001.

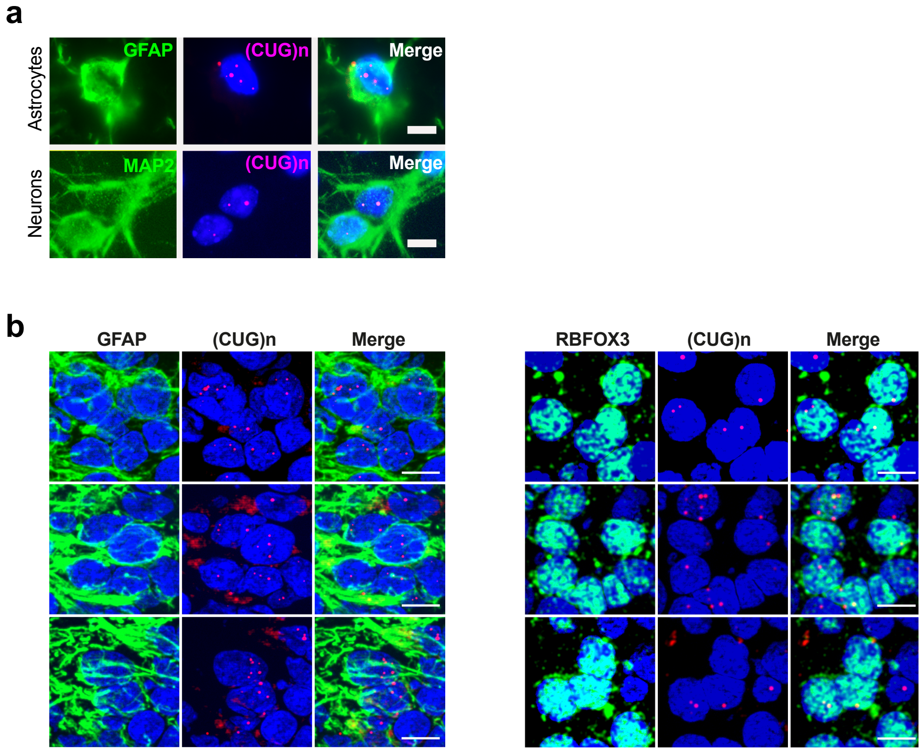

Supplementary Fig. 11. Foci accumulation in DM1 human brains. (a) FISH analysis of nuclear foci accumulation in human frontal cortex. Representative GFAP-expressing astrocytes and MAP2-posive neurons are shown. Scale bar, 10 µm. **(b)** RNA FISH combined with protein immunofluorescence, illustrating greater foci accumulation in GFAP-expressing-astrocytes, relative to RBFOX3/NeuN-expressing neurons. Scale bar, 10 µm. The experiments were performed on tissue samples from 3 DM1 individuals.

Supplementary Tables

Supplementary Table 1. RNA sequencing of primary DMSXL brain cells versus primary WT control cultures.
Data uploaded onto the GEO database.
GEO accession: GSE162093.
https://www.ncbi.nlm.nih.gov/geo/query/acc.cgi?&acc=GSE162093

Supplementary Table 2. Summary of transcript expression changes in DMSXL astrocytes.

Gene	Expression (RPM)		Log₂ fold change	$p_{adjusted}$[b]	Protein function
	WT	DMSXL			
Fbxl7[a]	208.5±16.7	22.8±5.5	-3.2	3.6E-34	Component of a E3 ubiquitin protein complex [1]
Rnf130	2217.0±64.9	1586.0±17.9	1.7	6.4E-06	E3 ubiquitin protein ligase [2]
Col15a1	35.2±1.5	110.7±15.4	-0.48	8.5E-09	Structural protein of the extracellular matrix [3]
Vsir (4632428N05Rik)	950.0±28.9	712.6±17.2	-0.41	1.2E-03	Immunoregulatory receptor [4]
Mov10	748.2±28.8	550.2±13.6	-0.44	5.1E-03	RNA helicase. miRNA processing [5]

[a]Transgene integration site. *Fbxl7* gene expression is disrupted by the human transgene. [b]Wald test adjusted for multiple comparisons, using the procedure of Benjamini and Hochberg.

Supplementary Table 3. Genes selected for missplicing validation in primary DMSXL astrocytes. Genes are distributed by the three top categories enriched in primary DMSXL astrocytes. GO ontology terms associated with each category are categorized into one of the 3 ontologies. BP, biological process; CC, cellular component; MF, molecular function.

Category	Accession ID	Name	Ontology	Genes for validation
**Cell adhesion**	GO:0048041	Focal adhesion assembly	BP	*Clasp1*<br>*Dmd*<br>*Fermt2*<br>*Itga6*<br>*Itgb4*<br>*Mpdz*<br>*Sorbs1*
	GO:0010810	Regulation of cell-substrate adhesion	BP	
	GO:0005913	Cell-cell adherens junction	CC	
	GO:0005925	Focal adhesion	CC	
	GO:0050839	Cell adhesion molecule binding	MF	
**Cytoskeleton**	GO:0031110	Regulation of microtubule polymerization or depolymerization	BP	*Clasp1*<br>*Fermt2*<br>*Inf2*<br>*Numa1*
	GO:0005874	Microtubule	CC	
	GO:0008017	Microtubule binding	MF	
	GO:0015631	Tubulin binding	MF	
	GO:0008092	Cytoskeletal protein binding	MF	
**Plasma membrane**	GO:0099072	Regulation of postsynaptic membrane neurotransmitter receptor levels	BP	*Capzb*<br>*Clasp1*<br>*Dmd*<br>*Fermt2*<br>*Mpdz*<br>*Numa1*
	GO:0099738	Cell cortex region	CC	
	GO:0030027	Lamellipodium	CC	
	GO:0030165	PDZ domain binding	MF	

Supplementary Table 4. Primary antibodies for western blotting and immunofluorescence

Protein	Supplier	Catalogue #	RRID	Application
ALDH1L1	Arigo	ARG10691	N/A	WB
CELF1	Millipore	05-621	AB_309851	WB
CELF2	Sigma	C9367	AB_1078584	WB
GAD65/GAD67	Millipore	ABN904	AB_2893025	IF
GPHN	Synaptic systems	147011	AB_887717	IF
GFAP	Abcam	Ab7260-50	AB_305808	WB, IF
GJA1	Abcam	Ab11370	AB_297976	WB, IF
GM130	BD Biosciences	610822	AB_398141	IF
HOMER1	Synaptic systems	160003	AB_887730	IF
MAP2	Santa Cruz Biotechnology	sc-80013	AB_1126218	IF
MBNL1	GE Morris; Oswestry, UK	MB1a(4A8)[a]	AB_2618248	WB, IF
	Abcam	Ab45899[b]	AB_1310475	WB
MBNL2	GE Morris; Oswestry, UK	MB2a(3B4)[c]	AB_2618250	WB, IF
	Abcam	Ab105331[d]	AB_10862122	WB
RBFOX3/NeuN	Abcam	Ab104225	AB_10711153	IF
S100B	Sigma	S2644	AB_477501	IF
SOX9	R&D Systems	AF3075	AB_2194160	IF
TUBB3	Covance	PRB-435P-100	AB_291637	WB
VCL (Vinculin)	Cell Signaling Technology	4650	AB_10559207	WB
	Sigma-Aldrich	V9131	AB_477629	IF
VGLUT1	Synaptic systems	135511	AB_887879	IF

RRID, Research Resource Identifier; N/A, not applicable; WB, western blot; IF, immunofluorescence. [a]Rabbit antibody raised against recombinant full-length human MBNL1 protein (epitope: VPSL, amino acids 140-143). [b]Rabbit antibody raised against a synthetic peptide corresponding to human MBNL1 amino acid sequence 250-350. [c]Rabbit antibody raised against recombinant full-length human MBNL2 protein (epitope: PYLA, amino acids 132-135). [d]Rabbit antibody raised against a synthetic peptide corresponding to human MBNL2 amino acid sequence 46-95.

Supplementary Table 5. Oligonucleotide primer sequences for qRT-PCR analysis.

Gene	Oligonucleotide primer sequences
Gfap	5’-GCTAACGACTATCGCCGCCAAC-3’<br>5’-TCCAGCCGAGCAAGTGCCTC-3’
Gpr17	5’-TCACAGCTTACCTGCTTCCC-3’<br>5’-GACCGTTCATCTTGTGGCTCT-3’
Mbnl1	5’-CGGGAC ACA AAA TGG CTAAC-3’<br>5’-TTGCAGTTCTCTCTGGAGCA-3’
Mbnl2	5’-CCCAAAAGTTGCCAGGTTGA-3’<br>5’-AGTGTGTCGGAGGATGAAGA-3’
Polr2a	5’-GGCTGTGCGGAAGGCTCTG-3’<br>5’-TGTCCTGGCGGTTGACCC-3’
Syp	5’-CCCCCTTCCTTCTCTCCCTCTG-3’<br>5’-CCATCTCCTCTCCACCCATTTCATC-3’

Supplementary Table 6. Oligonucleotide primer sequences for the splicing analysis of mouse alternative exons.

Gene	Alternative exon[a]	Size (bp)	Oligonucleotide primer sequences
Capzb	11	113	5'-GCACGCTGAATGAGATCTACTTTG-3'<br>5'-CCGGTTAGCGTGAAGCAGAG-3'
Clasp1	28	24	5'-TGACCTGGAAGCAGCAGTGG-3'<br>5'-CCGCAGATAATGGGGAATGC-3'
Dmd	81	32	5'-TCCCCAGGACACAAGCACAG-3'<br>5'-CCATCGCTCTGCCCAAATC-3'
Fermt2	4a	36	5'-GCTTGAGCTGGAAGGACCTCTTATC-3'<br>5'-GCAGAAGTTGGTGACAAAGGGC-3'
Fn1	33	270	5'-CCACTGTGGAGTACGTGGTTAGTG-3'<br>5'-TGGGTGTCACCTGACTGAACTTC-3'
Inf2	22	57	5'-CTGAAGATACCCCGGATGCC-3'<br>5'-CCGACGAGAGCACTCACTTG-3'
Itga6	27	130	5'-GGGATTCTGATGCTGGCTCTATTAG-3'<br>5'-GGCTTTGGGTAGTGTGAGGTGTTC-3'
Itgb4	35	162	5'-CAGGGTGGAGAAGACTACGAAAAC-3'<br>5'-GGTTAGGGATGTTGAGCCGATG-3'
Mbnl1	7	54	5'-GCTGCCCAATACCAGGTCAAC-3'<br>5'-TGGTGGGAGAAATGCTGTATGC-3'
Mbnl2	9	54	5'-ACCGTAACCGTTTGTATGGATTAC-3'<br>5'-CTTTGGTAAGGGATGAAGAGCAC-3'
Mbnl2	11	36	5'-GTCTTCAACCCCAGCGTCTT-3'<br>5'-AGCCTTAGGGTTGTGGTCTG-3'
Mpdz	28	99	5'-TGAACAAGCTGTGGAAGCCA-3'<br>5'-GGAGGGAGGAACATTGCACA-3'
Numa1	16	42	5'-GACCCACTTGGCTGAAATGC-3'<br>5'-GTCAGCTTCTTACTTAGTTCTTCC-3'
Sorbs1	6	90	5'-CTGCATCTGGGAAGACTCGCCT-3'<br>5'-GACTTGCTTTCATGCTTCGGAGATTC-3'
Sorbs1	27	168	5'-CCAGCTGATTACTTGGAGTCCACAGAAG-3'<br>5'-GTTCACCTTCATACCAGTTCTGGTCAATC-3'

[a] Alternative exons are numbered according to the FasterDB web interface (http://fasterdb.ens-lyon.fr/faster/home.pl)

Supplementary Table 7. Oligonucleotide primer sequences for the splicing analysis of human alternative exons.

Gene	Alternative exon[a]	Size (bp)	Oligonucleotide primer sequences
CAPZB	10b	113	5'-GCACCGCCCATTACAAGTTG-3'<br>5'-AGCCTCCACCAGGTCATTCTTC-3'
CLASP1	27	108	5'-CGCTAAAGTGGTTTCACAGTCCC-3'<br>5'-TTCTGCTACATCCTCAGTCTGCCG-3'
DMD	85	32	5'-TCCCCAGGACACAAGCACAG-3'<br>5'-CATCGCTCTGCCCAAATCATC-3'
FERMT2	4a	33	5'-TGACCAGTCTGAAGATGAGGCAC-3'<br>5'-GCTTGAGGCTTGAACATTTTTGC-3'
INF2	22	57	5'-GGAAGCGAAGGAAGAAGCGTC-3'<br>5'-GGTTGCCTTTAGGAAGCAGGTG-3'
ITGA6	27	130	5'-GAGTGACTGTGTTTCCCTCAAAGAC-3'<br>5'-CAGCCACGCCAAAAATAAAGG-3'
ITGB4	35	159	5'-CCGCTCAGAACACTCACACTCG-3'<br>5'-TGGTTGGGCAGGAGGTCTTC-3'
MPDZ	28	99	5'-CCATTCGGAAAGCAGGCAAC-3'<br>5'-TTCGGCAAAGGCTGAAGGAG-3'
NUMA1	20	42	5'-GTATGAGGGTGCCAAGGTCAAG-3'<br>5'-TGGTCAGAGTCAGCCAGTTTCTTAC-3'
POLR2A	N/A	N/A	5'-AGAGAAGCTGGTGCTCCGTA-3'<br>5'-AGCGCAGGAAGACATCATCA-3'
SORBS1	6	96	5'-AGTTGCAGACGACTTGTCCTGC-3'<br>5'-CCCTTCCCAGTGCAGATTTTTG-3'
	30	168	5'-CTGGGGATCTCACTAGCTTGGAG-3'<br>5'-GCCGTGGTGTTCTCCTTCATAC-3'

[a] Alternative exons are numbered according to the FasterDB web interface (http://fasterdb.ens-lyon.fr/faster/home.pl)

Supplementary References

1. Liu, Y. *et al.* The Proapoptotic F-box Protein Fbxl7 Regulates Mitochondrial Function by Mediating the Ubiquitylation and Proteasomal Degradation of Survivin. *Journal of Biological Chemistry* **290**, 11843–11852 (2015).

2. Guais, A. *et al.* h-Goliath, paralog of GRAIL, is a new E3 ligase protein, expressed in human leukocytes. *Gene* **374**, 112–120 (2006).

3. Myllyharju, J. & Kivirikko, K. I. Collagens, modifying enzymes and their mutations in humans, flies and worms. *Trends in Genetics* **20**, 33–43 (2004).

4. Lines, J. L. *et al.* VISTA Is an Immune Checkpoint Molecule for Human T CellsVISTA Negatively Regulates Human T Cells. *Cancer Research* **74**, 1924–1932 (2014).

5. Gregersen, L. H. *et al.* MOV10 Is a 5′ to 3′ RNA Helicase Contributing to UPF1 mRNA Target Degradation by Translocation along 3′ UTRs. *Molecular Cell* **54**, 573–585 (2014).

Original uncropped scans of blots and gels

Figure 1

Panel 1b

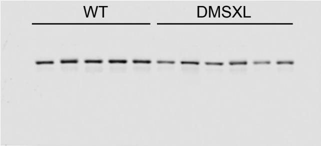

GFAP, Frontal cortex

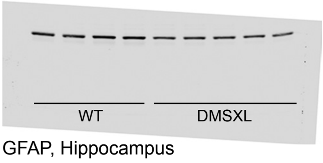

GFAP, Hippocampus

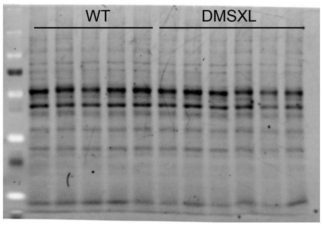

Total protein, Frontal cortex

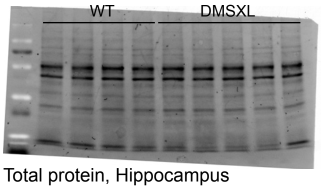

Total protein, Hippocampus

Figure 4

Panel 4c

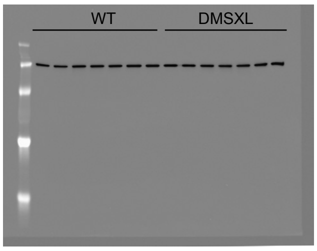

Vinculin, Primary Astrocytes

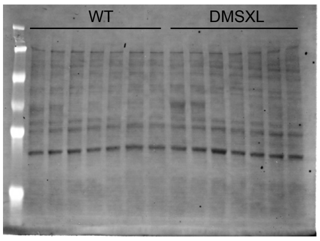

Total protein, Primary Astrocytes

Figure 6

Panel 6d

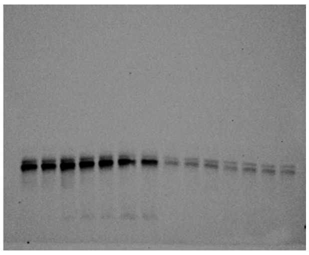

MBNL1, Primary astrocytes

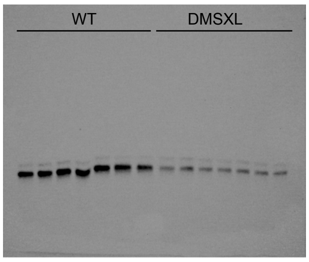

MBNL1, Primary Neurons

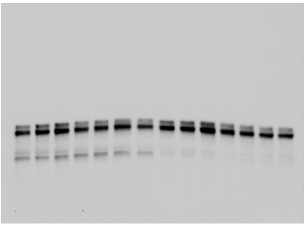

MBNL2, Primary astrocytes

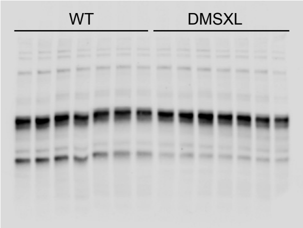

MBNL2, Primary Neurons

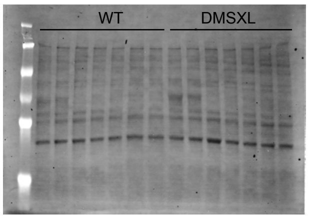

Total protein, Primary astrocytes

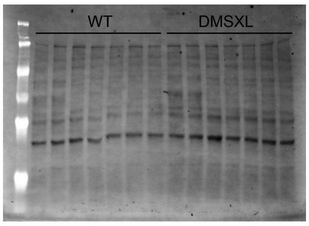

Total protein, Primary Neurons

Panel 6e

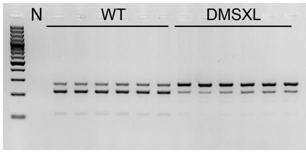

Mbnl1 exon 7
Primary astrocytes
N = No cDNA control

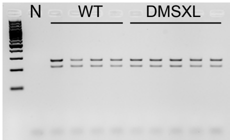

Mbnl1 exon 7
Primary neurons
N = No cDNA control

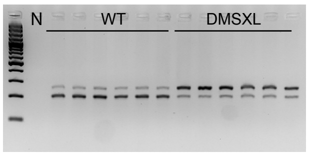

Mbnl2 exon 9
Primary astrocytes
N = No cDNA control

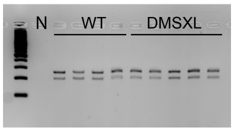

Mbnl2 exon 9
Primary neurons
N = No cDNA control

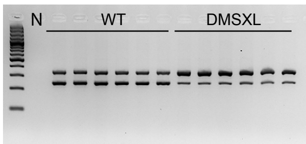

Mbnl2 exon 11
Primary astrocytes
N = No cDNA control

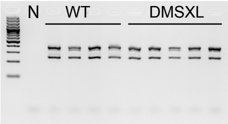

Mbnl2 exon 11
Primary neurons
N = No cDNA control

Figure 7

Panel 7b

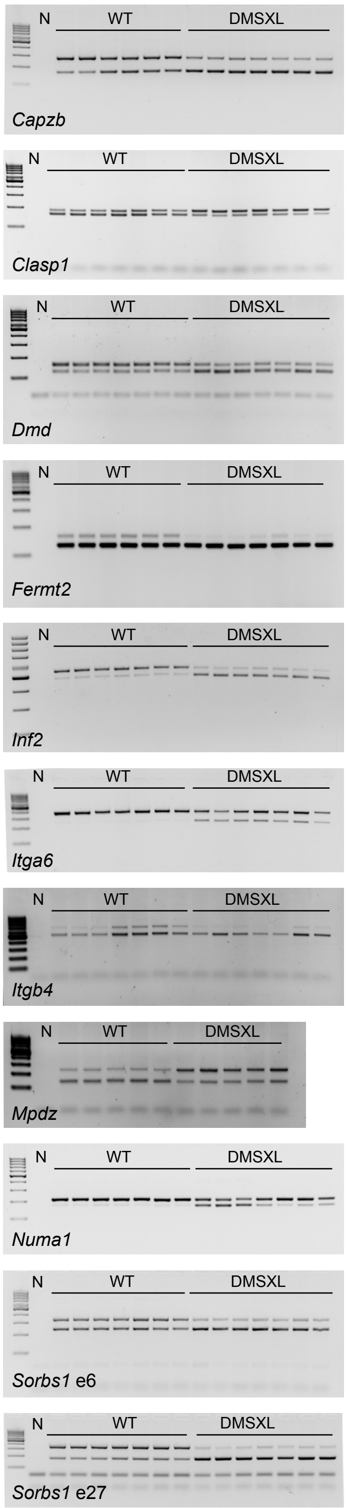

N = No cDNA control
T = Tissue cDNA control

Panel 7e

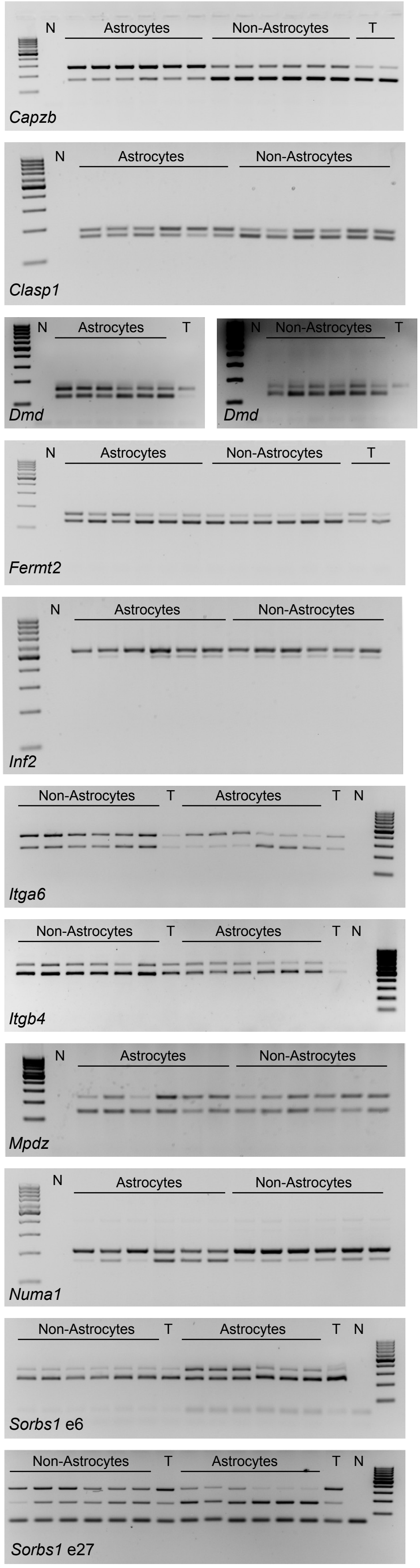

N = No cDNA control
T = Tissue cDNA control

Figure 8

Panel 8a

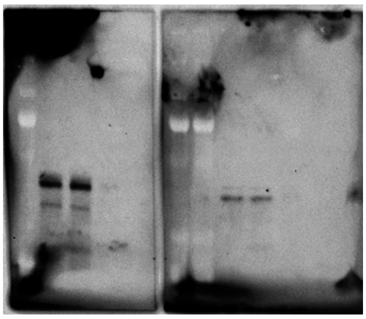

MBNL1 MBNL2

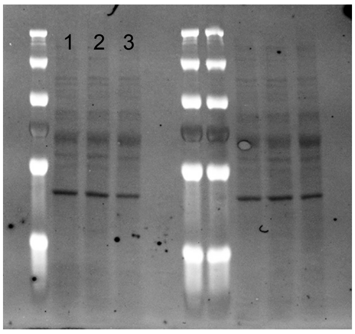

Total protein

Panel 8d

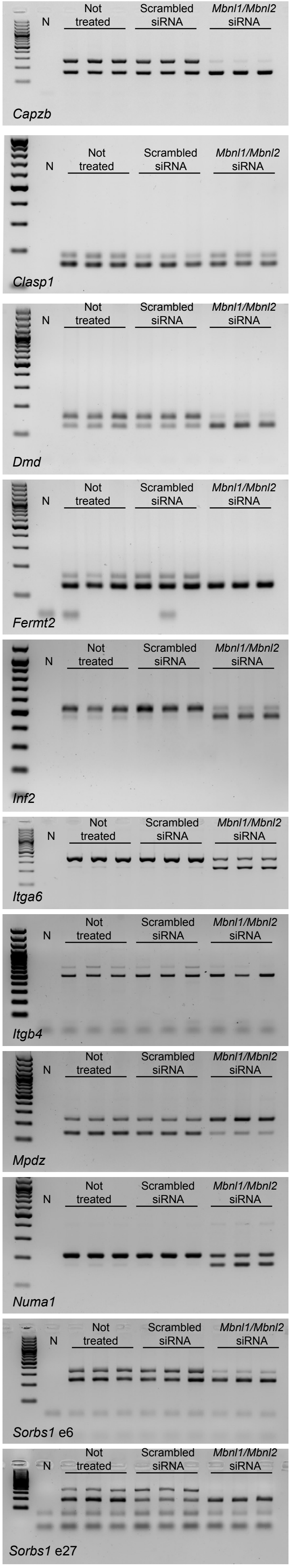

N = No cDNA control

Figure 9

Panel 9c

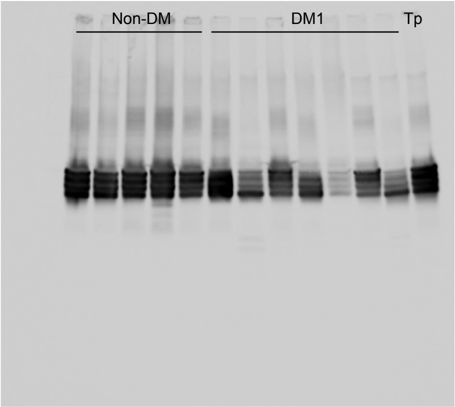

GFAP
Human frontal cortex
Tp = Temporal cortex, excluded from analysis

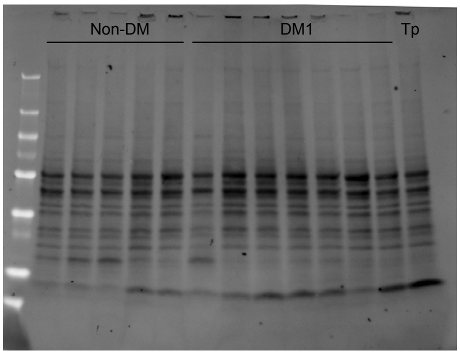

Total protein
Human frontal cortex
Tp = temporal cortex, excluded from analysis

Panel 9d

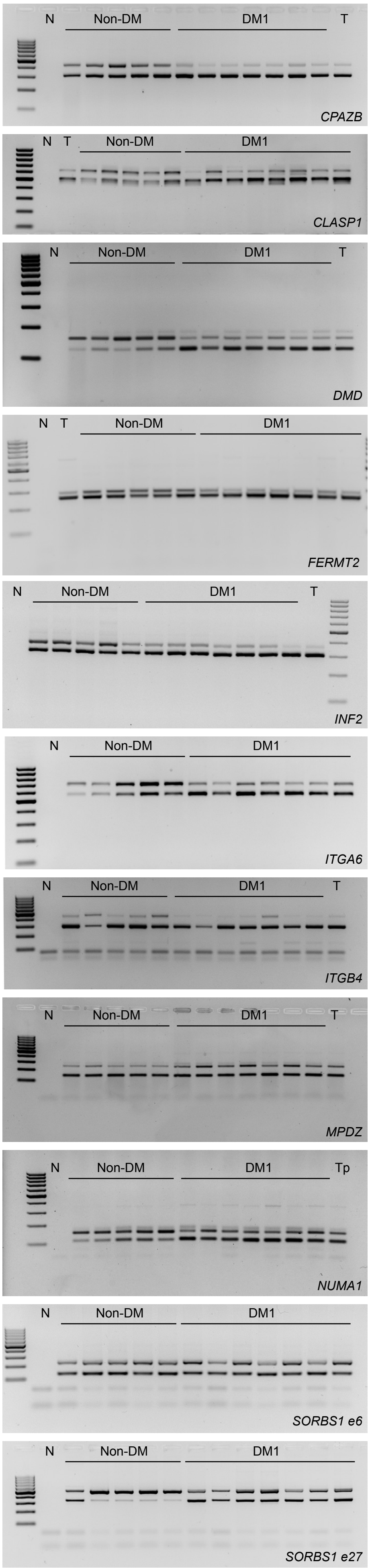

N = No cDNA control
T = Brain tissue cDNA control

Panel 9e

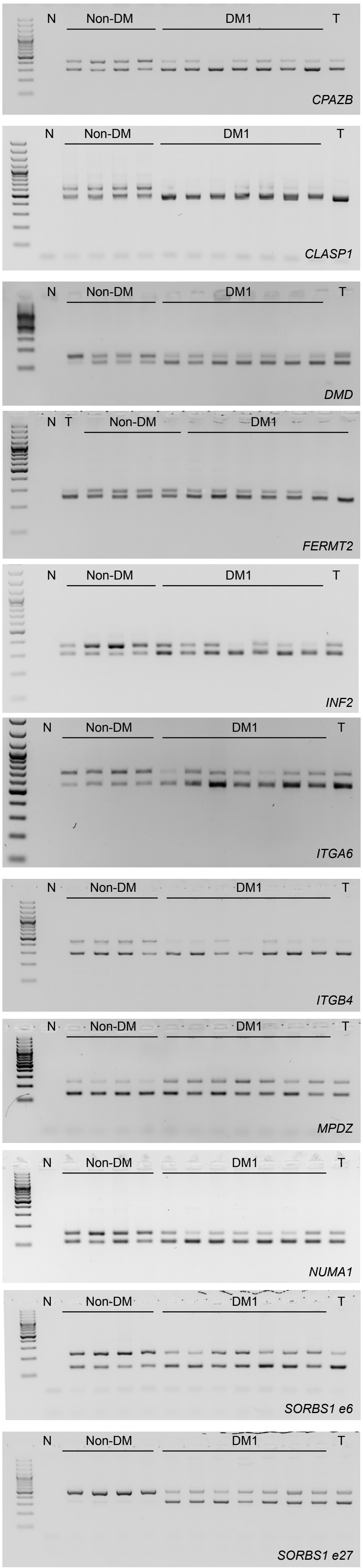

Supplementary Figure 9

Panel S9a

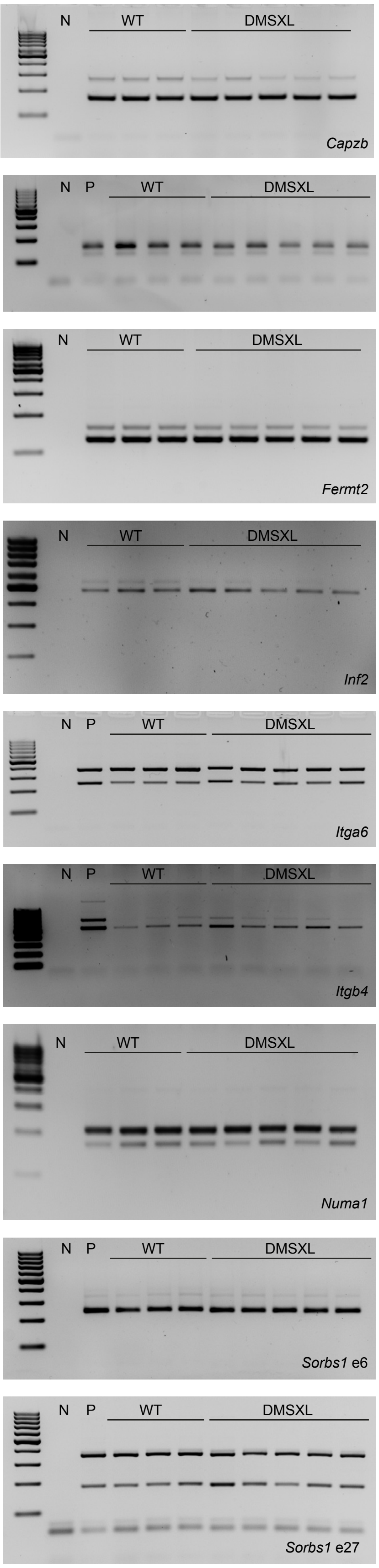

N = No cDNA control
P = cDNA pool, positive control

Panel S9b

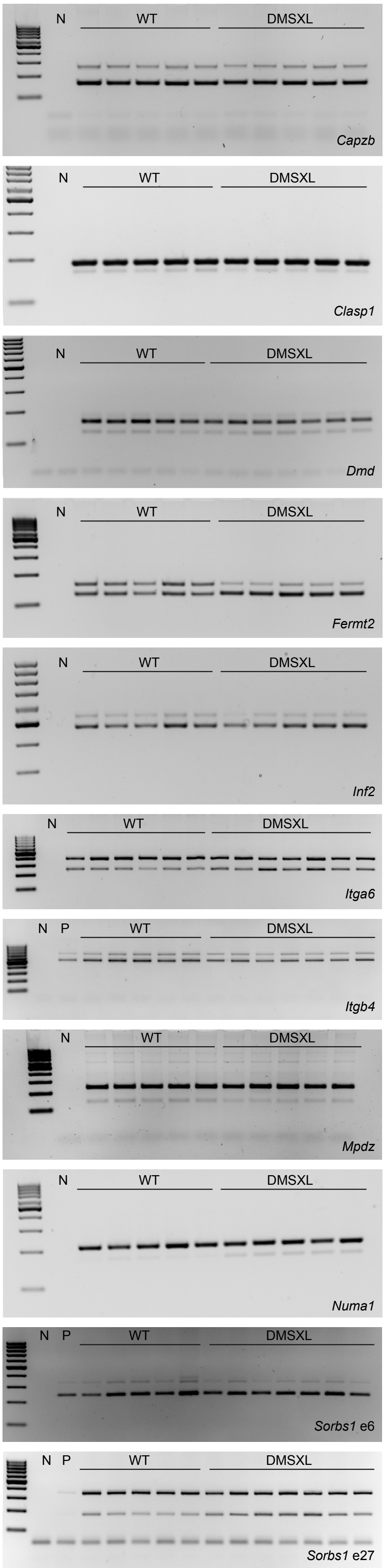

N = No cDNA control
P = cDNA pool, positive control

Panel S9c

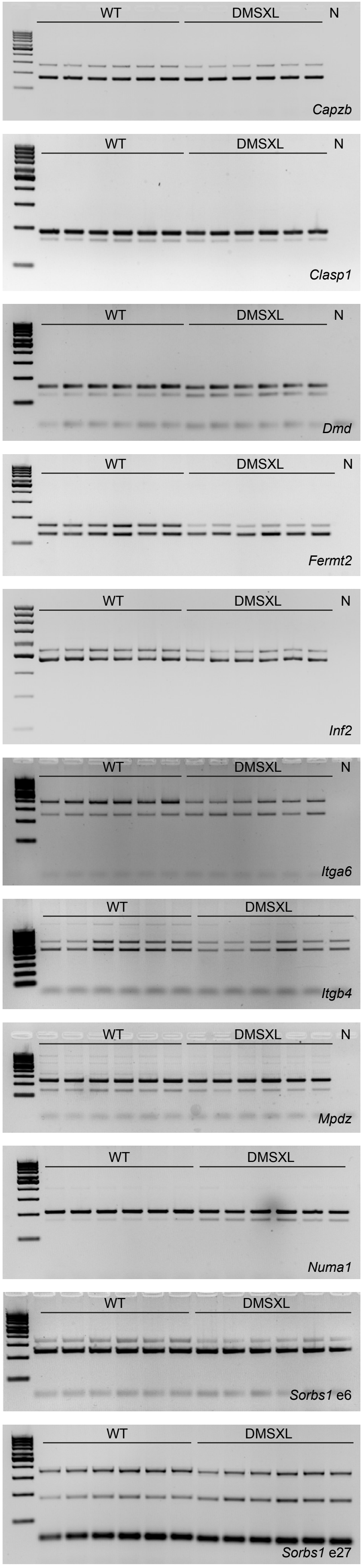

N = No cDNA control
P = cDNA pool, positive control

Lorem ipsun

Supplementary Figure 9

Panel S9d

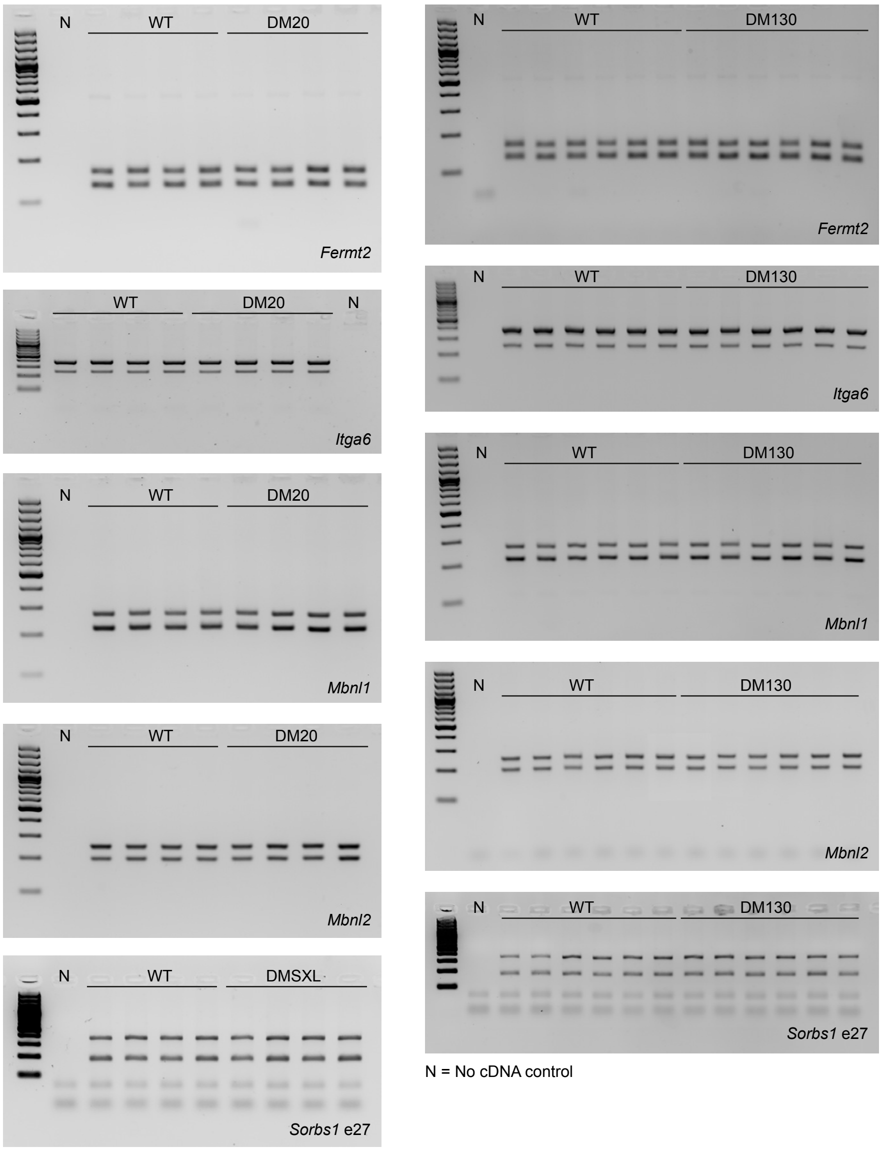

N = No cDNA control

Panel S9e

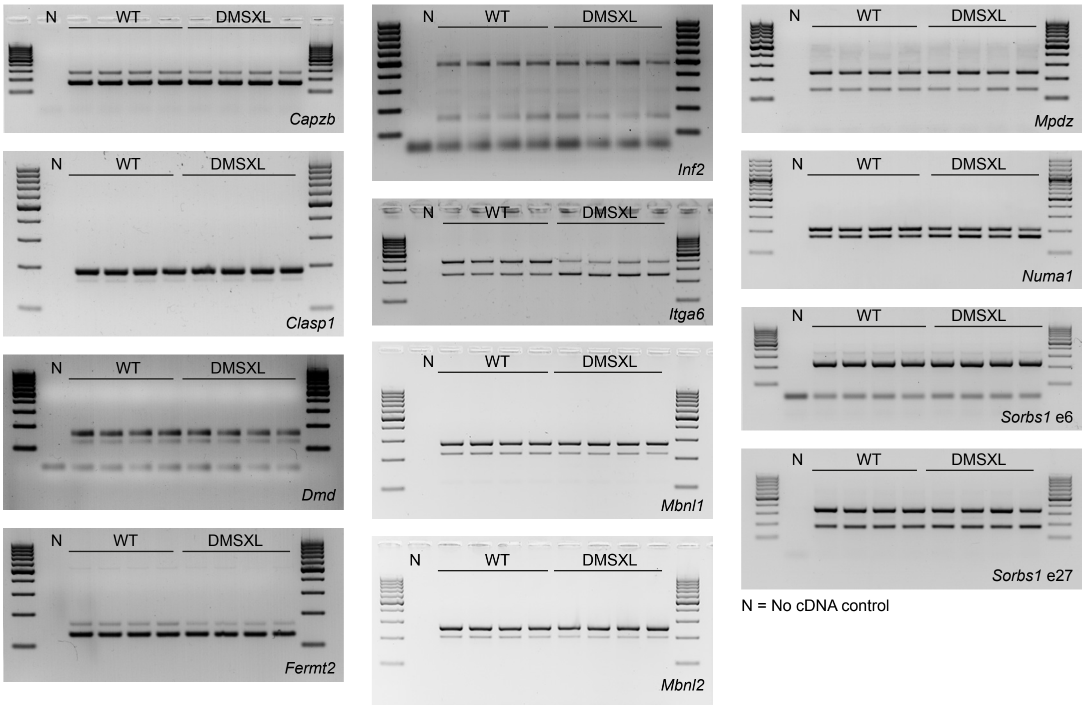

N = No cDNA control

Supplementary Figure 9

Panel S9f

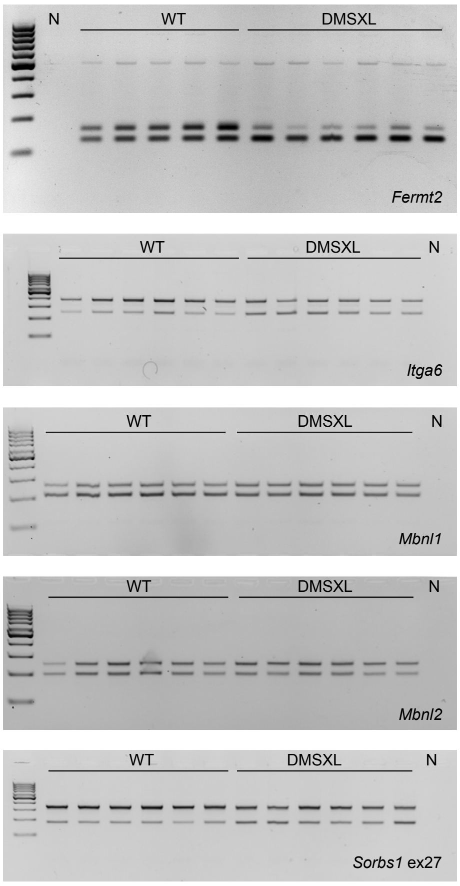

N = No cDNA control

Panel S9g

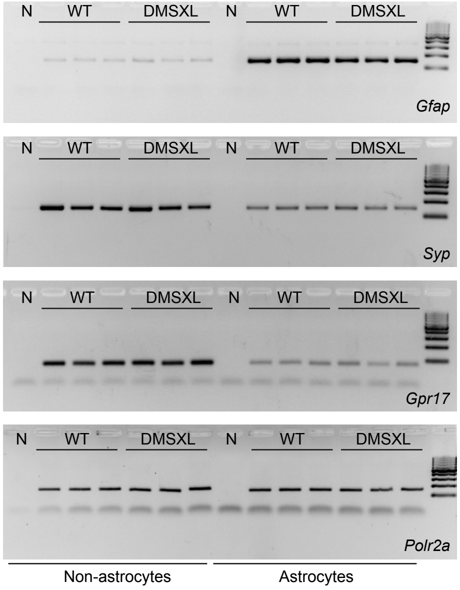

N = No cDNA control

Supplementary Figure 10

Panel S10a

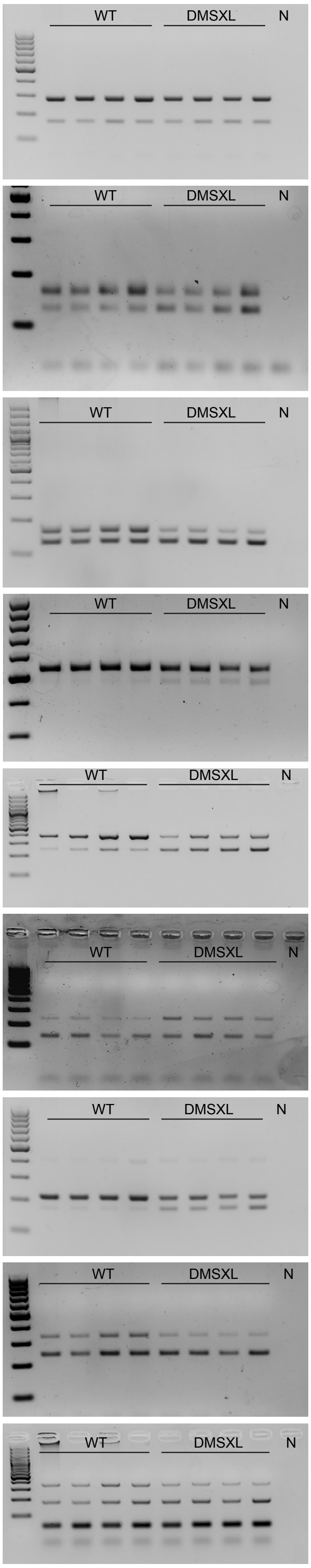

N = No cDNA control

Panel S10b

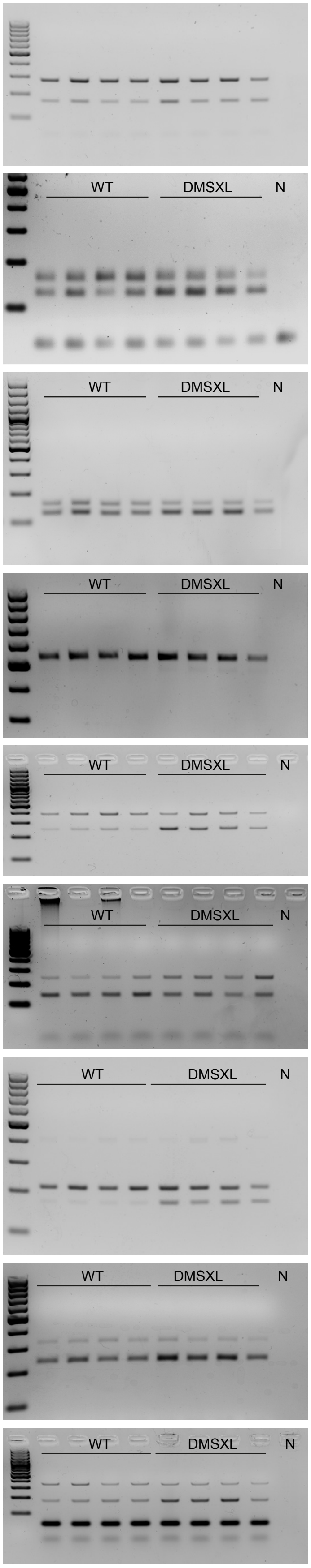

N = No cDNA control

Panel S10c

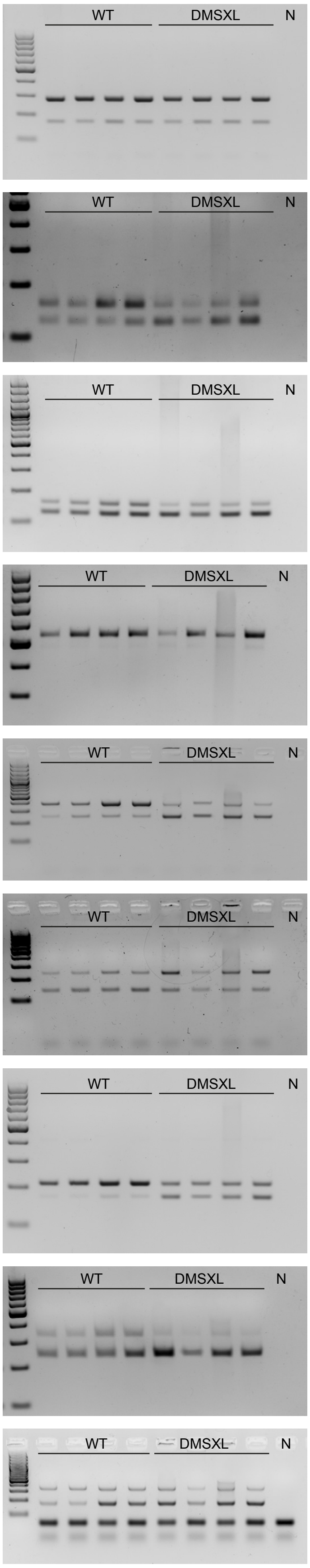

N = No cDNA control
