# Supplementary material for: Myotonic dystrophy RNA toxicity alters morphology, adhesion and migration of mouse and human astrocytes
Source: Nat Commun. 2022 Jul 4;13:3841. doi: 10.1038/s41467-022-31594-9 (PMC9253038; doi:10.1038/s41467-022-31594-9)
Supplement: Supplementary file 3 — Reporting Summary [file 41467_2022_31594_MOESM3_ESM.pdf]

nature research

Corresponding author(s): Mário Gomes-Pereira; Geneviève Gourdon

Last updated by author(s): Jun 3, 2022

Reporting Summary

Nature Research wishes to improve the reproducibility of the work that we publish. This form provides structure for consistency and transparency in reporting. For further information on Nature Research policies, see our Editorial Policies and the Editorial Policy Checklist.

Statistics

For all statistical analyses, confirm that the following items are present in the figure legend, table legend, main text, or Methods section.

n/a	Confirmed	
☐	☒	The exact sample size (*n*) for each experimental group/condition, given as a discrete number and unit of measurement
☐	☒	A statement on whether measurements were taken from distinct samples or whether the same sample was measured repeatedly
☐	☒	The statistical test(s) used AND whether they are one- or two-sided<br>*Only common tests should be described solely by name; describe more complex techniques in the Methods section.*
☐	☒	A description of all covariates tested
☐	☒	A description of any assumptions or corrections, such as tests of normality and adjustment for multiple comparisons
☐	☒	A full description of the statistical parameters including central tendency (e.g. means) or other basic estimates (e.g. regression coefficient) AND variation (e.g. standard deviation) or associated estimates of uncertainty (e.g. confidence intervals)
☐	☒	For null hypothesis testing, the test statistic (e.g. *F*, *t*, *r*) with confidence intervals, effect sizes, degrees of freedom and *P* value noted<br>*Give P values as exact values whenever suitable.*
☒	☐	For Bayesian analysis, information on the choice of priors and Markov chain Monte Carlo settings
☒	☐	For hierarchical and complex designs, identification of the appropriate level for tests and full reporting of outcomes
☒	☐	Estimates of effect sizes (e.g. Cohen's *d*, Pearson's *r*), indicating how they were calculated

Our web collection on statistics for biologists contains articles on many of the points above.

Software and code

Policy information about availability of computer code

Data collection	xCELLigence RTCA System and Software (Agilent), v1.2.1<br>IncuCyte ZOOM Basic Analysis Tool (Essen BioScience), v2016A<br>Incucyte Cell Migration Assay Kit (Essen BioScience), v2016A<br>IncuCyte NeuroTrack Analysis Module (Essen BioScience), v2016A<br>ChemiDoc Imaging System and the ImageLab TouchSoftware (Bio-Rad), v2.4.0.03.<br>LI-COR Odyssey CLx15 Imaging System and Software (Licor), v5.2<br>Simple Western JESS Compass Software (ProteinSimple), v2.2.3.<br>Leica Application Suite X Software (Leica), 4.13.0
Data analysis	Fiji - ImageJ software (https://imagej.net/downloads), v2.1.0.<br>FlowJo Software (BD Biosciences), v9.9.6.<br>IncuCyte NeuroTrack Analysis Module (Essen BioScience), v2016A<br>DESeq2 software package (open source), v1.16.1.<br>FARLINE pipeline (http://kissplice.prabi.fr/pipeline_ks_ farline), v1<br>Webgestalt Tool (http://www.webgestalt.org/), v2019<br>Image Lab Software (Bio-Rad), v6.1.0.<br>Image Studio Lite (Licor), v5.2.5.<br>Prism (GraphPad Software, Inc), v8 and v9<br>Simple Western JESS Compass Software (ProteinSimple), v2.2.3.

For manuscripts utilizing custom algorithms or software that are central to the research but not yet described in published literature, software must be made available to editors and reviewers. We strongly encourage code deposition in a community repository (e.g. GitHub). See the Nature Research guidelines for submitting code & software for further information.

Data

Policy information about availability of data

All manuscripts must include a data availability statement. This statement should provide the following information, where applicable:

- Accession codes, unique identifiers, or web links for publicly available datasets
- A list of figures that have associated raw data
- A description of any restrictions on data availability

The authors declare that all data supporting the findings of this study are available within the paper and its supplementary information files.
All the RNA sequencing data sets that were used as input for the study are available at GEO (Gene Expression Omnibus). GEO accession: GSE162093.
Direct link: https://www.ncbi.nlm.nih.gov/geo/query/acc.cgi?&acc=GSE162093

Field-specific reporting

Please select the one below that is the best fit for your research. If you are not sure, read the appropriate sections before making your selection.

☒ Life sciences ☐ Behavioural & social sciences ☐ Ecological, evolutionary & environmental sciences

For a reference copy of the document with all sections, see nature.com/documents/nr-reporting-summary-flat.pdf

Life sciences study design

All studies must disclose on these points even when the disclosure is negative.

Sample size	No sample size calculation was performed for this study. Sample size was chosen based on previous experience and historical data collected in the laboratory for each individual assay [Huguet et al. (2012) PLoS Genetics, 8: e1003043; Hernández-Hernández et al. (2013) Brain, 136: 957; Sicot et al. (2017) Cell Reports, 19: 2718; González-Barriga (2021) Frontiers in Cellular Neuroscience, 15: 125].<br>A minimum of 3 biological replicates, up to a maximum of 7 were studied, to account for variability between samples.
Data exclusions	Out of the MIO-M1 biological replicates studied in each group (CTG-expansion cell cultures versus no-expansion control cultures), 1 replicate was excluded from each group, because the cells failed to grow in culture.<br>Out of the four independent astrocyte cultures derived from 4 different DM130 newborns did not grow. This biological replicate was excluded from the analysis.<br>Out of the five independent astrocyte cultures transfected with Mbnl1/Mbnl2 siRNA or scrambled siRNA, one did not grow. Non-transfected control cultures derived from the same embryo did not grow either. This replicate was excluded from the analysis.<br>No additional data were excluded from the experiments and analyses presented.
Replication	All experiments were reproduced to reliably support conclusions stated in the manuscript. Real-time live cell monitoring (conductance, confluence, migration, neuritogenesis in co-culture) were repeated at least 3 times per biological replicate (i.e. primary cell culture derived from individual embryos). Immunofluorescence staining was performed at least 3 times per biological replicate. Western blot and RT-PCR splicing analysis were performed at least twice per biological replicate. Sholl morphological analysis was perform once per biological replicate (individual animal).
Randomization	Our study is not subject to randomization since it does not involve allocation of cell cultures, animals of participants into experimental groups. As a result randomization is not relevant for the experiments performed.
Blinding	The investigator was blind to the genotypes and groups of mouse tissue samples being tested in western blot and RT-PCR analysis. The investigator was blind to the cell groups being tested in MBNL1/MBNL2 knocking down experiments.<br>When handling animals, blinding was not possible, because experienced investigators can distinguish transgenic animals from wild-type controls, given the difference in body weight. For the same reasons, the investigator was not blind to the genotype of primary cell cultures (astrocytes and neurons), as well as MIO-M1 cell groups.

Reporting for specific materials, systems and methods

We require information from authors about some types of materials, experimental systems and methods used in many studies. Here, indicate whether each material, system or method listed is relevant to your study. If you are not sure if a list item applies to your research, read the appropriate section before selecting a response.

Materials & experimental systems

n/a	Involved in the study
☐	☒ Antibodies
☐	☒ Eukaryotic cell lines
☒	☐ Palaeontology and archaeology
☐	☒ Animals and other organisms
☐	☒ Human research participants
☒	☐ Clinical data
☒	☐ Dual use research of concern

Methods

n/a	Involved in the study
☒	☐ ChIP-seq
☐	☒ Flow cytometry
☒	☐ MRI-based neuroimaging

Antibodies

Antibodies used

ALDH1L1 (Arigo, ARG10691); Annexin V-FITC (BD Horizon, 556547); BrdU-FITC (BD Horizon, 347583); CELF1 (Millipore, 05-621); CELF2 (Sigma, C9367); GAD65/GAD67 (Millipore, ABN904); GPHN (Synaptic systems, 147011); GFAP (Abcam, Ab7260-50); GJA1 (Abcam, Ab11370); GM130 (BD Biosciences, 610822); HOMER1 (Synaptic systems, 160003); MAP2 (Santa Cruz Biotechnology, sc-80013); MBNL1 (GE Morris; Oswestry, UK; clone MB1a(4A8)); MBNL2 (GE Morris; Gswestry, UK; MB2a(clone 3B4)); RBFOX3/NeuN (Abcam, Ab104225); S100B (Abcam, Ab52642); SOX9 (R&D Systems, AF3075); TUBB3 (Covance, PRB-435P-100); VCL (Cell Signaling Technology, 4650); VCL (Sigma-Aldrich, V9131); VGLU1 (Synaptic systems; 135511); HRP-labelled anti-mouse (Protein Simple, DM-002); HRP-labelled anti-rabbit (Protein Simple, DM-001).

Validation

ALDH1L1 (Arigo, ARG10691). Tested reactivity: human, mouse, rat, bovine, horse and pig. Tested applications: ICC/IF, IHC-Fr, WB.
Annexin V-FITC (BD Horizon, 556547). Tested reactivity: mouse. Tested applications. Flow cytometry, intracellular staining (flow cytometry). 7 references on the manufacturer's website.
BrdU-FITC (BD Horizon, 347583). Tested reactivity: human, mouse, rat and many other species. Tested applications. Flow cytometry, intracellular staining (flow cytometry). 7 references on the manufacturer's website.
CELF1 (Millipore, 05-621). Tested reactivity: human, mouse, rat, rabbit, pig. Tested applications: EMSA, IP, WB, ICC. 4 references on the manufacturer's website.
CELF2 (Sigma, C9367). Tested reactivity: human, mouse, chicken. Tested applications: ICC, IP, WB. 7 references on the manufacturer's website.
GAD65/GAD67 (Millipore, ABN904). Tested reactivity: human, mouse, rat. Tested applications: WB, IHC-P. 17 references on the manufacturer's website.
GPHN (Synaptic Systems, 147011). Tested reactivity: human, mouse, rat, pig, goldfish, zebrafish, chicken. 173 references on the manufacturer's website.
GFAP (Abcam, Ab7260-50). Tested reactivity: human, mouse, rat. Tested applications: WB, IHC-P, ICC. 557 references on the manufacturer's website.
GJA1 (Abcam, Ab11370). Tested reactivity: human, mouse, rat. hamster, cow, dog, pig, monkey. Tested applications: WB, ICC, IHC-P, IHC-Fr. 229 references on the manufacturer's website.
GM130 (BD Biosciences, 610822). Tested reactivity: human, mouse, rat, dog. Tested applications: WB, IF, IP. 5 references on the manufacturer's website.
HOMER1 (Synaptic Systems, 160003). Tested reactivity: human, mouse, rat. Tested applications: WB, IP, ICC, IHC, EM. 47 references on the manufacturer's website.
MAP2 (Santa Cruz Biotechnology, sc-80013). Tested reactivity: human, mouse, rat. Tested applications: WP, IP, IF. 4 references on the manufacturer's website.
MBNL1 (GE Morris; Oswestry, UK; clone MB1a(4A8)). Tested reactivity: human, mouse. Tested applications: WB, IF, IHC, ELISA. Original publication: doi: 10.1111/j.1365-2443.2007.01112.x.
MBNL1 (Abcam, Ab45899). Tested reactivity: human. Predicted reactivity: mouse, rat. Tested applications: WB, IHC/IF, IHC. 10 references on the manufacturer's website.
MBNL2 (GE Morris; Gswestry, UK; MB2a(clone 3B4)). Tested reactivity: human, mouse. Tested applications: WB, IF, IHC, ELISA. Original publication: doi: 10.2353/ajpath.2009.080520.
MBNL2 (Abcam, Ab105331). Tested reactivity: human. Predicted reactivity: Mouse, Rat, Rabbit, Horse, Chicken, Guinea pig, Cow, Cat, Dog, Zebrafish. Tested applications: WB. 2 references on the manufacturer's website.
RBFOX3 (Abcam, Ab10711153). Tested reactivity: human, mouse, rat. Tested applications: WB, IHC/IF. 171 references on the manufacturer's website.
S100B (Sigma, S2644). Tested reactivity: human, guinea pig, rat, bovine. Predicted reactivity: Mouse. Tested applications: IHC-P. 73 references on www.citeab.com.
SOX9 (R&D Systems, AF3075). Tested reactivity: human, mouse, rat. Tested applications: WB, IHC. 51 references on the manufacturer's website.
TUBB3 (Covance, PRB-435P-100). Tested reactivity: human, mouse, rat. Tested applications: WB, IHC-P, ICC. 70 references on the manufacturer's website.
VCL (Cell Signaling Technology, 4650). Tested reactivity: human, mouse, rat, monkey, dog. Tested applications: WB. 80 references on the manufacturer's website.
VCL (Sigma-Aldrich, V9131). Tested reactivity: human, mouse, rat, dog, chicken, turkey, frog, cow. Tested applications: IHC-Fr, IF, WB. 1081 references on the manufacturer's website.
VGLUT1 (Synaptic Systems, 135511). Tested reactivity: mouse, rat. Tested applications: WB, IP, ICC, IHC-Fr, IHC-P. 29 references on the manufacturer's website.

Eukaryotic cell lines

Policy information about cell lines

Cell line source(s)	Original MIO-M1 cell line obtained from UCL Institute of Ophthalmology, Prof. Stephen Moss.
Authentication	The cell line was not authenticated.
Mycoplasma contamination	All cell lines used in our study tested negative for mycoplasma contamination. qPCR detection of mycoplasma contamination was routinely performed (every 3-6 months)
Commonly misidentified lines (See ICLAC register)	No commonly misidentified cell lines were used in this study.

Animals and other organisms

Policy information about studies involving animals; ARRIVE guidelines recommended for reporting animal research

Laboratory animals	Species: Mus musculus.<br>Strain: >99% C57BL/6 background.<br>Sex of animals: Both male and female mice were studied.<br>Age of animals for neuron collection: embryonic day E16.<br>Age of animals for astrocyte collection: newborn mice at postnatal day 1.<br>Age animals for the analysis of splicing, protein expression and astrocyte phenotypes in vivo: P10, 1 month and 4 monhs.<br>Animals were maintained on a 12-h light/dark cycle with food and water ad libitum. Temperature: 22 +/- 2°C. Humidity: 40-60%.
Wild animals	The study did not involve wild animals.
Field-collected samples	The study did not involve samples collected from the field.
Ethics oversight	The study protocol was approved by Prefecture de Police (Paris) and the French Veterinary Department. Authorization for animal experimentation number 75 003. Animal facility approval number B 91 228 107.

Note that full information on the approval of the study protocol must also be provided in the manuscript.

Human research participants

Policy information about studies involving human research participants

Population characteristics	DM1 patients.<br>Genotype/diagnosis: DM1, CTG repeat expansion detected by Souther blot or PCR amplification.<br>Age: Mean, 64.3; SD, 5.1<br>Age of onset: mean, 46,0; SD, 12.5 (all patients presented adult onset clinical forms)<br>Sex: 3 males, 4 females<br>Cause of death: pneumonia or heart failure.<br><br>Non-DM controls<br>Clinical diagnosis: No clinical diagnosis (2 individuals), Charcot-Marie Tooth Disease (1 individual), Rheumathoide arthrisis (1 individual), Limb-Girdle muscular dystrophy (1 individual).<br>Genotype: Absence of DM1 or DM2 repeat expansion.<br>Age: Mean, 68.6; SD, 10.4<br>Sex: 4 males, 1 female<br>Cause of death: pneumonia or heart failure.
Recruitment	Adult individuals were recruited at Asahikawa Medical Center (Japan) and Okayama University (Japan). Informed consent was obtained from all patients or relatives.<br>Post-mortem brain tissue was collected from adult DM1 patients with confirmed genetic diagnosis (Southern blot detection or PCR amplification of expanded CTG trinucleotide repeat expansion). Non-DM controls were recruited to match the age and sex of DM1 patients, whenever possible.<br>Since all the patients fulfilled the genetic diagnosis criteria, we believe there was no selection bias in our results.
Ethics oversight	All experiments using human samples were approved by the local Ethics Committees of the host institutions where brain samples were collected: Asahikawa Medical Center (Japan) and Okayama University (Japan). Written informed consent of specimen use for research was obtained from all patients.

Note that full information on the approval of the study protocol must also be provided in the manuscript.

Flow Cytometry

Plots

Confirm that:

☒ The axis labels state the marker and fluorochrome used (e.g. CD4-FITC).

☒ The axis scales are clearly visible. Include numbers along axes only for bottom left plot of group (a 'group' is an analysis of identical markers).

☒ All plots are contour plots with outliers or pseudocolor plots.

☒ A numerical value for number of cells or percentage (with statistics) is provided.

Methodology

Sample preparation	For cell cycle analysis, primary mouse astrocytes were arrested by serum starvation. Following cell cycle re-entry in serum-containing medium, cells were incubated 10 µM bromodeoxyuridine, trypsinized, fixed in 70% ethanol and stained with anti-BrdU-FITC and 3 µM propidium iodide. 10,000 cells were analysed per embryo.<br>For the analysis of cell death, mouse primary astrocytes were analyzed after O/N incubation with DMSO or 0.5 µM Staurosporine. Cells were trypsinized, stained 5% Annexin V-FITC to detect apoptotic cells, PI to detect necrotic cells and 2,5% Cd11b-V450 to exclude microglia contamination. Staining was performed for 15 min prior to the analysis of 10,000 cells per embryo.
Instrument	MACSQuant Flow Cytometer from Miltenyi Biotec
Software	FlowJo from BD (Becton, Dickinson and Company)
Cell population abundance	Cells were not sorted for the purposes of these experiments. Cells were gated to determine the percentage of cells in each phase of the cell cycle, and to quantify cell death by apoptosis and necrosis.
Gating strategy	After forward versus side scatter (FSC vs SSC) gating to exclude cell debris, cell cycle phases were considered as follows: G1-phase cells were BrdU negative with low PI staining (200 intensity units), S-phase cells are BrdU positive and show a range of low to high PI intensity (200 to 400 intensity units) and G2/M cells are BrdU negative with high PI intensity (400 intensity units).<br>First forward versus side scatter (FSC vs SSC) gating allowed exclusion of cell debris, then ACSA2 positive, Cd11b negative astrocytes were gated, on which the percentage of Annexin V-positive, PI-positive astrocytes was calculated.

☒ Tick this box to confirm that a figure exemplifying the gating strategy is provided in the Supplementary Information.
